# Challenges with using names to link digital biodiversity information

**DOI:** 10.3897/BDJ.4.e8080

**Published:** 2016-05-25

**Authors:** David Patterson, Dmitry Mozzherin, David Peter Shorthouse, Anne Thessen

**Affiliations:** ‡ University of Sydney, Sydney, Australia; § Illinois Natural History Survey, Champaign, IL, United States of America; | Canadian Museum of Nature, Ottawa, Canada; ¶ The Data Detektive, Waltham, United States of America; # The Ronin Institute for Independent Scholarship, Montclair, United States of America

## Abstract

The need for a names-based cyber-infrastructure for digital biology is based on the argument that scientific names serve as a standardized metadata system that has been used consistently and near universally for 250 years. As we move towards data-centric biology, name-strings can be called on to discover, index, manage, and analyze accessible digital biodiversity information from multiple sources. Known impediments to the use of scientific names as metadata include synonyms, homonyms, mis-spellings, and the use of other strings as identifiers. We here compare the name-strings in GenBank, Catalogue of Life (CoL), and the Dryad Digital Repository (DRYAD) to assess the effectiveness of the current names-management toolkit developed by Global Names to achieve interoperability among distributed data sources. New tools that have been used here include Parser (to break name-strings into component parts and to promote the use of canonical versions of the names), a modified TaxaMatch fuzzy-matcher (to help manage typographical, transliteration, and OCR errors), and Cross-Mapper (to make comparisons among data sets). The data sources include scientific names at multiple ranks; vernacular (common) names; acronyms; strain identifiers and other surrogates including idiosyncratic abbreviations and concatenations. About 40% of the name-strings in GenBank are scientific names representing about 400,000 species or infraspecies and their synonyms. Of the formally-named terminal taxa (species and lower taxa) represented, about 82% have a match in CoL. Using a subset of content in DRYAD, about 45% of the identifiers are names of species and infraspecies, and of these only about a third have a match in CoL. With simple processing, the extent of matching between DRYAD and CoL can be improved to over 90%. The findings confirm the necessity for name-processing tools and the value of scientific names as a mechanism to interconnect distributed data, and identify specific areas of improvement for taxonomic data sources. Some areas of diversity (bacteria and viruses) are not well represented by conventional scientific names, and they and other forms of strings (acronyms, identifiers, and other surrogates) that are used instead of names need to be managed in reconciliation services (mapping alternative name-strings for the same taxon together). On-line resolution services will bring older scientific names up to date or convert surrogate name-strings to scientific names should such names exist. Examples are given of many of the aberrant forms of ‘names’ that make their way into these databases. The occurrence of scientific names with incorrect authors, such as chresonyms within synonymy lists, is a quality-control issue in need of attention. We propose a future-proofing solution that will empower stakeholders to take advantage of the name-based infrastructure at little cost. This proposed infrastructure includes a standardized system that adopts or creates UUIDs for name-strings, software that can identify name-strings in sources and apply the UUIDs, reconciliation and resolution services to manage the name-strings, and an annotation environment for quality control by users of name-strings.

## Introduction

The ‘big new biology’ complements traditional and reductionist approaches to biological research because it will be based on open sharing of data that will enable co-operative enterprises and large scale projects (National Research Council of the National Academies 2009). Within this emerging area, names are said to have a special role (Patterson et al. 2010; Pyle 2016) because, from the time of Linnaeus, biologists have applied a convention of forming and using scientific names. Scientific names annotate almost all useful biological statements for most of the intervening 250 years. They still play that role, but are supplemented increasingly with records in which organisms are identified through molecular sequence data - such as molecular barcodes (Hebert et al. 2003, Federhen et al. 2016). Names act as a system of metadata with which we can organize open distributed data in a biologically meaningful way, and as such they make larger scale studies possible. Projects such as LifeWatch, Atlas of Living Australia and especially Encyclopedia of Life rely on names to organize content (Fuentes and Fiore 2014, Patterson 2010). The Global Names Architecture is a vision to make a names-based cyberinfrastructure available for open and free use. Along with phylogenetic informatics (​Parr et al. 2012), molecular bioinformatics, ecoinformatics (Michener and Jones 2012), and ontologies (Bard and Rhee 2004), a names-based cyberinfrastructure will make possible collaborative projects that extend across the scope and scale of biology, and create new opportunities for discovery.

The use of names as metadata present an array of problems. They include incorrectly formed names, changes to the correct name for a taxon, or the use of the same name-string (the sequence of characters, digits, and spaces that makes up the name) for more than one taxon (Patterson et al. 2010). This has led to a collection of environments and tools (see Peng et al. 2012 for overview) and standard names-lists (e. g. Zermoglio et al. 2016) to help manage the use of names both as metadata and to prevent the use of names that are mis-spelled or no longer are endorsed as the correct name by any taxonomic authorities. The Global Names Architecture (GNA) is a vision for an underlying free and open names-based cyber-infrastructure that will provide services (such as confirming spelling, authority informationm or indicating if the name has been rendered into synonymy) to users of names by drawing on expert sources of taxonomic and nomenclatural knowledge and adding value to them with new tools and data management environments. Some of the GNA tools are included in this study, but not all contingencies have been addressed, nor are all tools developed to deliver production grade (aiming at 95% satisfaction) services as yet. GNA aims to be dynamic (see below) and to embrace differing views as to the correct name for a taxon - views that are held in ‘Taxonomic Authority Files’ (van den Berghe et al. 2015).

The most significant known challenge with the use of names as metadata is the ‘many names for one taxon’ problem (Patterson et al. 2010). Because of it, a search initiated with a single name-string may not find content that applies to a taxon because it was labelled with a different name. This problem has many causes. One is if species are moved to a different genus. The cryptophyte known as *Chilomonas paramecium* was moved to *Cryptomonas* when the species *paramecium* was found to have a sister group relationship with species within *Cryptomonas* (Hoef-Emden and Melkonian 2008). In response, the species was moved to *Cryptomonas* and a second name, *Cryptomonas paramecium*, was created for the same species. The new name is a homotypic (objective or nomenclatural) synonym of the first name (see glossary of terms). That is, additional names are created for the same taxon because of new taxonomic and phylogenetic insights.

A second cause of a species having more than one name is when improved taxonomic awareness demonstrates that two species that were described independently turn out to be the same species. *Triactinomyxon gyrosalmo* and *Myxosoma cerebralis* are different life-history stages of the same species, a discovery that came long after the stages were described as separate species (Wolf and Markiw 1984). The two names are heterotypic (taxonomic or subjective) synonyms. The issue of two ‘species’ being found to be different stages in the life history of a single species is common among fungi that have sexual (teleomorph) and asexual (anamorph) reproductive stages; *Hypocrea jecorina* and *Trichoderma reesei* are scientific names for the teleomorph and anamorphic states of a single fungus species, respectively.

Third, not all taxonomists agree about everything all of the time. More than one name may be endorsed for the same taxon at the same time by different taxonomists. *Drosophila melanogaster* and *Sophophora melanogaster* are alternate scientific names for the same species of fruit-fly but reflect different taxonomic preferences.

Fourth, the name-strings for a species may not be forms of scientific names. Scientific names are presented in a latinized form, are compliant with the relevant code of nomenclature, or, if the codes do not apply to them (for example, because they are names of high ranking taxa), they are written in a comparable form consistent with the expectations of biologists. Scientific names may include annotations, authors, and dates of nomenclatural acts. Code-compliance typically addresses the names of families, genera, subgenera, species and subspecies. This definition of scientific names is not consistent with the use of the same term by GenBank (see "Results" below). Other classes of 'names' include common names, also referred to as vernacular or colloquial names, are part of living languages, such as French, Tagalog, or Latvian. Another class of 'names' are surrogates that may be strain numbers, acronyms, or other strings that take the place of a name. Finally, taxa may be distinguished using identifiers in the form of short molecular sequences or barcodes, or with data identifiers such as LSIDs or UUIDs. This classification is inexact as, illustrated below, some name-strings include scientific elements, or may be part common names, part acronym, or part surrogate (here and elsewhere, examples of name-strings from this exercise are presented in **bold**):

- **Balaenoptera acutorostrata dwarf minke whale**
- **Saccharomyces cerevisiae Red Star baking yeast**
- **Platycheirus punctulata group sp. BOLD:AAL9445**
- **Staphylococcus sp. S2IP4(2011)**
- **Diaporthales sp. nwa_besc_246k**
- **Platygyra cf. verweyi DH-2010.**

Any name-string may be mis-spelled, distorted because of OCR errors, inadvertently concatenated, or have alternate spellings. Some examples of these problems are: **arex appropinquata Schum.** for **Carex appropinguata**; **Troglodyted troglodyted** for **Troglodytes troglodytes**; **Verena mulinoides Speg.** for **Verbena mulinoides Spegazzini, 1902**. **Kummerovia striata** and **Kummerowia striata** are alternative spellings in the same source; and **corbulasulcata** is a concatenation of **Corbula sulcata**. Another source of problems is the intrusion of non-code-compliant characters that can create additional name-strings due to encoding problems. To be compliant with nomenclatural codes, scientific names should - usually - use the English version of Latin. Yet, the following characters occur in name-strings that were presented as scientific names and are indexed by the Global Names Index.

!"#$%&'()*+,-./0123456789:;<=>?@ABCDEFGHIJKLMNOPQRSTUVWXYZ[\]^_`abcdefghijklmnopqrstuvwxyz{|}~ € ‚ƒ„…†‡ˆ‰Š‹Œ Ž ‘’“”•–—˜™š›œ žŸ¡¢£¤¥¦§¨©ª«¬­®¯°±²³ ´µ¶·¸¹º»¼½¾¿ÀÁÂÃÄÅÆÇÈÉÊËÌÍÎÏÐÑÒÓÔÕÖ×ØÙÚÛÜÝÞßàáâãäåæçèéêëìíîïðñòóôõö÷øùúûüýþÿāăąĆćĉČčĎďđēĕėęěğīĭİıĶĹĺĽľŁłńŅņňŌŏŐőŒœŕŘřŚśŜŞşŠšţťũūŭůűŸŹźŻżŽžſƒǎǔǧǾȘșȚțȳˆ˙˚˜́̈ΑΒΗΘΛΦΨΩαβγδεζθικλνАВРСФалосуखठपमषुृ्কঘমলােỳ ​‎‐‑–—‘’‚“”„†‡•…‰‹›⁄€™Ⅲ→∂−√∞∫≈≠≤≥◊♀ ㎜三乌二侧假光匙南原参变古叶味四团培大头姜少山峨川广早智术极林果根栽桂毛江波温牙狭白盈益眉眼短矮种穗竹紫细翅聚肾脉舞花苞苦莪菜蓝蔻西豆象郁金靖顶香黄龙가늬뢰리린먹무뿔싸쑤우할ﬀﬁ﻿＆（），：ｍ�

A different problem occurs when the same name is used for more than one taxon, that is, they are homonyms. The Codes of Nomenclature seek to prevent homonyms by stipulating that when a name is used, it can never be used again for another taxon. However, given the number of species and the absence of comprehensive nomenclators, it is possible that one author inadvertently introduces as a new name one that has already been used. *Dolium* was introduced in 1990 for an unusual euglenid (Larsen and Patterson 1990), but had already been used for a mollusc (Lamarck 1801). As different Codes of Nomenclature apply to different areas of life, the same name may be legitimately used for taxa of plants and of animals (*Peranema* is a name for a fern and for a flagellate). Up to 15% of generic names are homonyms (IRMNG homonyms, McNeill 1997), but the number of species-level homonyms (e.g. *Pieris japonica* Shirôzu, 1952 - a butterfly, and *Pieris japonica* (Thunberg) D. Don ex G. Don - a flowering plant) is small (a few hundred) (IRMNG species homonyms). In the absence of agreement to use a unified code, transregnal homonyms will need to be disambiguated to avoid information on unrelated species being included among the results of a search using a homonymic name. Reference to authors, species names in the case of generic homonyms; and taxonomic or other context has the potential to achieve disambiguation.

A further known problem arises with chresonyms. Scientific names may or may not include the names of the authors of the name, whereas chresonyms are references to scientific names as used by others (Smith and Smith 1972). The resulting name-string may have a special notation - such as a colon before the author - to indicate a usage of the name. Notations are often absent such that the chresonym name-string is indistinbguishable in form from a scientific name (with author). The name of the South American water willow usually ascribed to Nicolaus von Jacquin is included in Catalogue of Life with the five different authors: **Justicia carthagenensis Willd. ex Nees**, **Justicia charthaginensis L.** (purportedly a mis-spelling by Linnaeus), **Justicia carthaginensis Vahl**, **Justicia carthaginensis Jacq.** and **Justicia catharinensis Lindau** (a homonym, Flann, pers. comm.). The Plant List  has 5369 entries for fewer than 200 species of Rosa (Bruneau et al. 2007). Some taxonomists incorrectly include chresonyms within synonymy lists, but differences between synonnyms and chresonyms are often lost when names are gathered together from multiple sources.

Other known problems with the use of names as metadata relate to their inability to discriminate among taxonomic concepts (Remsen 2016). Concepts can be declared within a name-string by use of the terms ‘sec.’ or ‘sensu’ (Berendsohn 1995), but the meanings of concepts are rarely associated with the names, and we set this problem aside.

This paper draws on several sources of names to quantify the types of challenges presented in the use of names and to assess the extent of overlap. We emphasize issues relating to terminal taxa (species and infraspecies) because information associated with higher taxa has limited usefulness. Our intent is to identify the challenges that a names-based infrastructure will have to deal with in future biodiversity sciences disciplines (Hardisty and Roberts 2013). Given the time cost of this exercise where results have to be scrutinised by eye, we have not used many sources of data. A useful expension of this exercise would be to compare nomenclatural registries such as ZooBank, IPNI, and Index Fungorum with taxonomic compilations

## Materials and methods

In this paper we adopt the convention of using italics for the genus and species elements when we refer to a name as a scientific name (e.g. Carex scirpoidea Michx. ssp. convoluta (Kük.) Dunlop), but we use bold font when treating it as an example of a name-string that we need to manage (e.g. **Carex scirpoidea Michx. ssp. convoluta (Kük.) Dunlop**). All examples in the results of this paper are verbatim entries from the sources used in this study. The examples were copied from sources and pasted into this report.

A copy of the data is available from the DRYAD data repository (http://dx.doi.org/10.5061/dryad.50c71).

### Names sources

**Genbank.**

**Figure 1.**
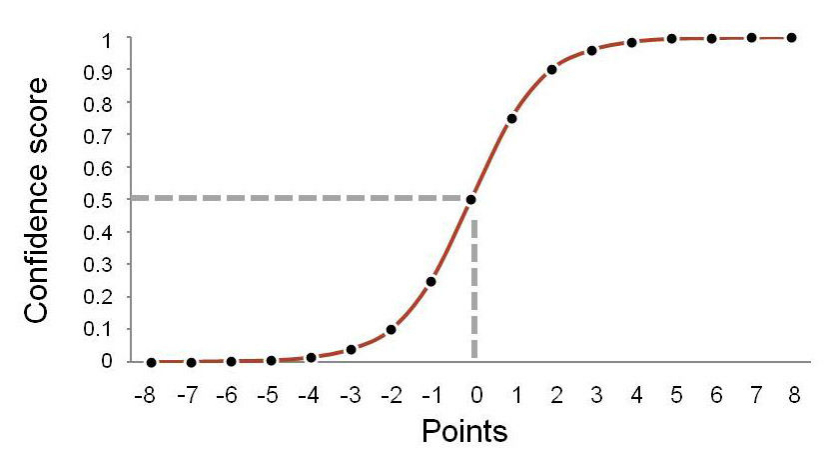
Sigmoid curve that converts the sum of positive and negative points that increase or decrease (respectively) into a confidence value. 0.5 indicates neutral confidence whereas 0.99 indicates high confidence.

GenBank taxonomy and names content has been described by Federhen (Federhen 2012, Federhen 2014). The names.dmp file was downloaded from GenBank (taxdump.zip at ftp://ftp.ncbi.nih.gov/pub/taxonomy) on 15th May 2015. The GenBank names.dmp file contained 1920102 records with four fields: (1) tax_id - the identifier of the node associated with this name, (2) name_txt - the name-string itself, but this is not guaranteed to be unique; (3) unique name - a variant name-string applied to a record if name_txt is not unique - for example **whiptail stingray <Dasyatis hawaiensis>** and **whiptail stingray <Dasyatis brevis>** disambiguate two meanings for **whiptail stingray**; and (4) name class - labels as indicated in Fig. 1. The name-strings are curated as evidenced by statements of synonymy, and provision of both scientific and colloquial names for non-terminal taxa all the way to all life (‘**Biota**’).

**Catalogue of Life** content was acquired on 25 July, 2015 using the DWCA export facility (Roskov Y 2015). It includes name-strings that relate to 1,606,554 species, 150,118 infraspecific taxa, 1,322,911 synonyms and 329,997 common names (http://www.catalogueoflife.org/annual-checklist/2015/info/ac). Different web pages provided by Catalogue of Life give slightly different numbers. CoL is believed to cover 70-84% of the (estimated) number of formally described species, drawing on the contributions of over 3,000 specialists. As the largest compilation of endorsed taxa, it offers a reference system that helps us to assess the level of interoperability that is achievable now and in the foreseeable future.

**DRYAD** (The Dryad Digital Repository, datadryad.org) is a repository for data underlying publications in evolution and ecology. It contains over 33,000 data files relevant to biodiversity. DRYAD is very flexible regarding data format and allows providers to decide what type of files to deposit. The DRYAD curation process does not include oversight of taxonomic names or name-strings and as a consequence the name-strings show considerable variation (see Results). To sample the name-strings in DRYAD, about 200 data packages were chosen randomly for download. Each data package included one or more data files. Unique name-strings (scientific names only) were identified in all data files by two human annotators (Kappa agreement = 0.832). If a taxon name was used as an adjective, such as in “crocodilian anatomy” it was not included in the lists. Mentions of genera were included as a separate reference to a taxon even if a species within that genus was mentioned.

The analysis also relied on content in **NameBank** (ubio.org) and **GNI** (gni.globalnames.org), uBio and Global Names repositories (respectively) of name-strings. GNI is seen as a ‘dirty’ bucket containing any name-string that was used as a label for a taxon. It currently has access to 24 million name-strings of which 17,275,622 are visible at gni.globalnames.org. The content of GNI has, to date, been rendered into 7,695,783 reconciliation groups using algorithms. GNI complements the cleaner buckets of name-strings from taxonomic compilations and nomenclatural registries.

### Software

The following software has been developed by the Global Names team, and is freely available (see globalnames.org).

**GN-UUID** (http://dx.doi.org/10.5281/zenodo.45036) creates UUID version 5 identifiers for name-strings (available at https://github.com/GlobalNamesArchitecture/gn_uuid/releases/tag/v0.5.0, see also http://globalnames.org/apps/gn-uuid/, http://globalnamesarchitecture.github.io/gna/uuid/2015/05/31/gn-uuid-0-5-0.html). UUID v5 is created using a SHA1 hash of a string in combination with a name space (https://www.ietf.org/rfc/rfc4122.txt), making it well suited for any form of name-string for taxa. As the UUID v5 is generated using information from the string, any environment will generate the same UUID as long as they agree on the generation of a name space. gn_uuid has a DNS domain “globalnames.org” defined as a name space. UUID v5 creates opportunities for the biodiversity community to mint uniform UUIDs for the same name-strings, associate them with their own data, and enable their information to be linked to other information on the same name-string.

**The ‘biodiversity’ Global Names parser** (http://dx.doi.org/10.5281/zenodo.45038) is a Ruby gem (https://github.com/GlobalNamesArchitecture/biodiversity/releases/tag/v3.4.1) (http://gni.globalnames.org/parsers/new) that takes incoming name-strings and divides them into their semantic components - such as genus name, species or subspecific epithets, author names, dates of nomenclatural acts, basionym author and date, annotations such as cf., nr, null, aff., ex., hybrid formulas and the like. The parser is able to distinguish the use of the term ‘**Bison**’ as a genus, species, and subspecies in the following examples:

- **Bison**
- **Bison bison**
- **Bison bison bison**
- **Bison bison athabascae**
- **Bos bison bison**

An updated version (http://parser.globalnames.org) is being described more fully elsewhere (Mozzherin et al., in press). The primary use of the parser is to transform a name-string into a canonical version. In so doing, it removes variation among versions of name-strings for the same taxon - as illustrated by the following variant forms of *Anolis barkeri* from CoL that are all rendered into the same canonical form **Anolis barkeri**:

- **Anolis barkeri**
- **Anolis barkeri (Schmidt, 1939)**
- **Anolis barkeri POWELL & BIRT 2001**
- **Anolis barkeri POWELL 2001**
- **Anolis barkeri Schmidt**
- **Anolis barkeri Schmidt 1939**
- **Anolis barkeri Schmidt, 1939**

Much of the variation among name-strings is associated with the authority information (for interesting examples see Kottelat 2015), which vary because author names may or may not be abbreviated, may be written out in different ways, may or may include punctuation, may or may not include dates, may have different styles of conversion from non-latin scripts, or may be replaced by the name and date of a usage of the name (i.e. the name-string is a chresonym). In the example above, **Anolis barkeri POWELL & BIRT 2001** and **Anolis barkeri POWELL 2001** are not code-compliant names and may be chresonyms that refer to the use of the name *Anolis barkeri* by Powell and by Powell and Birt. Apparent chresonyms may be created when a name is given of a subspecies or infraspecies with the appropriate authority but when the subspecific and/or infraspecific elements of the scientific name are removed.

The process of canonicalization involves parsing a name and then removing non-latinized and non-essential elements. One aim for this is to remove elements that show a lot of variation between lexical variants of the name-strings for the same species. The 'noisy' elements of a name include annotations or differences in author information. Onmce these are removed, differently presented versions of the same name found in different sources can be matched. There can be different versions of canonicalization. Complete canonicalization retains all of the latinized elements of the original name-string. Standard canonicalization retains only those elements that are required by the codes. The complete canonical of **Aaleniella (Danocythere)** is **Aaleniella (Danocythere)**, whereas the standard canonical of the same name is **Danocythere**. In this analysis we relied on standard canonicals.

**Ruby port (TaxaMatch fuzzy matcher).** Ruby Port fuzzy matcher (https://github.com/GlobalNamesArchitecture/taxamatch_rb/releases/tag/v1.1.1) is based on TaxaMatch (Rees 2014). It is a biologically informed spell-checker that seeks to identify variant spellings that may be caused by typographical, transliteration, or OCR errors. As a result, it can identify **Dorsophila melanogaster** as being a variant of **Drosophila melanogaster.** It combines the Damerau-Levenshtein distance algorithm with heuristic rules designed specifically for scientific names to produce improved levels of recall, precision and execution time. The number of actions such as a character change, addition, deletion within the source name-string that leads to a match with a target name-string is referred to as 'Edit Distance'. The greater the edit distance, the greater the level of tolerance that is required by Ruby Port to match names. The level of tolerance accepoted by the software can be adjusted.

**Global Names Cross-mapper** (https://github.com/GlobalNamesArchitecture/gn_crossmap) was developed in collaboration with Catalogue of Life as a means of making comparisons among lists of scientific names. It is a Ruby Gem (called gn_crossmap) (https://github.com/GlobalNamesArchitecture/gn_crossmap/releases/tag/v0.1.8, https://globalnamesarchitecture.github.io/gna/resolver/checklist/2015/05/11/gn-crossmap-gem.html) that cross-maps name-strings in a data source to the name-strings in another. The process can involve full name-strings or canonical names by invoking parsing tools. Cross-mapper can be applied to checklists that are supplied in CSV-form from, for example, spreadsheet environments such as MS Excel, Apple Numbers, Open Office, Libre Office, and Google Sheets. We include a ‘pre-processing’ step with regular expressions that can be used to eliminate recurring idiosyncrasies in sources of names to produce standardized names. The business rules of pre-processing can be adapted to suit each source. Pre-processing was used to manage the content from DRYAD in which there were a large number of name-strings that were created by concatenation of genus and species elements of the name interpolated with another character such as ‘_’. The tool is being described in more detail (Mozzherin et al., in prep.).

In this study, the names derived from GenBank and DRYAD were cross-mapped against Catalogue of Life. Each record in the source database was recorded as one of the following.

- Exact match: meaning that the full name-string in the source matched exactly a full name-string in Catalogue of Life.
- Canonical form exact match: the canonical form of the name-string in the source matched a canonical form of a name-string in Catalogue of Life.
- Partial canonical form match: part of the canonical form of the name-string matched a canonical string of a name-string within Catalogue of Life, this occurred when name-strings with subspecific or infrasubspecific elements matched to a canonical species binomen in Catalogue of Life. If no other match is found, the algorithm seeks to identify matches by genus only.
- Genus part match: If there is no partial match at the specific, or infraspecific levels, the algorithm tries to match the genus component of the name to genera present in the Catalogue of Life.
- Partial canonical form fuzzy match: A part of the canonical name (such as the genus of a binomen, or genus and species of a trinomen or polynomen) found a fuzzy (inexact) match to an element in Catalogue of Life.
- Canonical form fuzzy match 1: The canonical form of a name in the source matched a canonical name in Catalogue of Life with an Edit Distance of 1 (that is, a single manipulation such as a character change, addition, deletion of the source name-string would lead to an exact match with the target name-string).
- Canonical form fuzzy match 2 - 6: The canonical form of a name in the source matched a canonical name in Catalogue of Life with an Edit Distance of 2-6 (i.e. the source name-string would require 2-6 changes to match a name-string in Catalogue of Life).
- No match.

**Confidence**: We assign a confidence score to matches because even perfect matches may not be correct. In the case of homonyms, a source that uses the name *Aotus* may refer to a plant, but the match may be made to the identically spelled genus name for a monkey. Poor fuzzy matching may also be misleading. The name-string **Canela** can be fuzzily matched to the genus names **Canelo** or **Canala**, and from this information alone, we are unable to determine which is right. Matches of binomial or trinomial names, or of names with authorship information are more likely to be correct. Different authorships do not necessarily mean different taxonomic meaning. *Monochamus galloprovincialis* (Olivier, 1795) and *Monochamus galloprovincialis* Secchi, 1998 refer to the same species, the former including the original author of the basionym and the latter is a chresonym - a reference to the use of the name. The "confidence score" takes into account these issues.

The ‘score’ is achieved by adding or subtracting points for positive and negative features, and then converting the point score into a value between 0 and 1 using a sigmoid curve (Fig. 1). This follows the same principle as used by Boyle et al. (2013). The shape of the curve tends to exaggerate initial strong and weak features; but lessens the impact of additional features. Points reflect features of name-strings which increase the likelihood of a correct match, and negative values to features that decrease it. For example, an exact match of a uninomial genus name (**Erigeron**) adds one point to give a confidence score of 0.75; a match of a binomial name (**Erigeron altaicus** with **Erigeron altaicus Popov**) increases the probability significantly, for which we add 3 points to give a confidence score of 0.988. A match with all of the author information (**Erigeron annuus (L.) Pers.**) adds a further point to give a confidence score of 0.999. However, if the authorship of the name did not match (e.g. **Erigeron canadensis L.** with **Erigeron canadensis Brot.**) we subtract 2 points, to give an overall confidence score of 0.75. Results with scores of 0.5 and below need to be confirmed by a human check.

### Reclassification of name-strings

Cross-mapping of both DRYAD and GenBank name-strings to Catalogue of Life produced 1,988,845 results, greater than the number of original name-strings because some name-strings were mapped to more than one name in the target. The results were re-analysed by eye to categorise them in respect of their suitability to interconnect distributed data in a biologically meaningful fashion. The classes adopted are:

- **Clade identifiable species**: The name-string includes the name of a species but not any subordinate taxa; such name-strings may be used to interconnect content in distributed data environments. Included in this class are name-strings that are well formed and not well formed but from which the scientific name-string could be extracted (such as **Botryllus_planus_DQ346653** and **Hypothyris_anastasia_20507**).
- **Clade identifiable genus**: the name-string includes the name of a genus but no subordinate taxa; such name-strings have some value to interconnect content in distributed data environments, but they do so without full taxonomic detail. Included in this class are name-strings that were well formed, or not originally well formed but from which the scientific name-string could be extracted.
- **Clade identifiable infraspecies**: the name includes a species name and subordinate rank(s) such as subspecies, variety, form, or morph. Such name-strings may be used to interconnect content in distributed data environments. Included in this class are name-strings that were well formed, or were not originally well formed but from which the scientific name-string could be extracted.
- **Clade identifiable higher**: the name-string refers to a taxon with a rank higher than genus; such name-strings may interconnect content in distributed data environments but are taxonomically imprecise and have limited utility. Included in this class are name-strings that were well formed, or were not originally well formed but from which the scientific name-string could be extracted.
- **Common name**: A vernacular or colloquial name, matches based on common names may not be taxonomically precise.
- **Hybrids**: typically with two name-strings and the hybrid sign ‘x’, but also includes ‘natural hybrids’ with a single name-string and the hybrid sign.
- **Negated names**: Name-strings which include an annotation such as cf., nec., aff., nr, null or other comments to indicate that the scientific elements of the name-string in the record do not identify the taxon in question and should not be used to interconnect distributed content.
- **Not useful**: This category includes unresolvable acronyms, environmental samples without any taxonomic identity, name-strings that fuzzily match with edit distances greater than 2 (see results), abbreviated names, non-organismic molecules, some organelles, idiosyncratic forms of name-strings, and records of symbionts in which the host is named but the symbiont is not; none of the name-strings in this class can be used to link to other data sources.

### Data Resources

The data underpinning the analysis reported in this paper are deposited in the Dryad Data Repository at http://datadryad.org/submit?journalID=BDJ&manu=PJS_2_8080

## Results

The results are presented in subsections.

### GenBank Content

**Figure 2.**
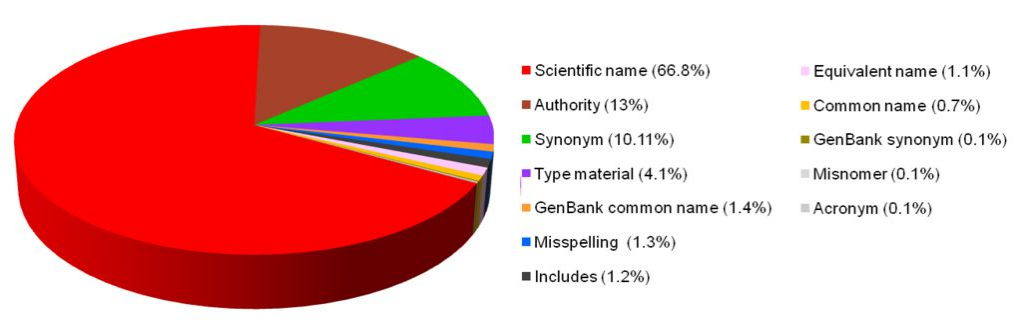
Profile of GenBank names.dmp file classified according to GenBank, to nearest 0.1%. (Anamorphs, GenBank anamorphs, Teleomorphs, GenBank acronyms, In-parts, and Blast names each account for less than 0.1% of GenBank content).

GenBank assigned its content of 1,920,102 name-strings to classes (Fig. 2​​). The quality of the GenBank classification is high, although some anomalies do occur and examples of them are illustrated below. The nature of GenBank classes is not always self-evident. As our concern is to address the interoperability achieved through name-strings associated with terminal taxa (species and infraspecies), our comments primarily relate to terminal taxa. We comment on the GenBank classes, as they informed our approach to reclassifying them appropriate to the objectives of this study.

**Acronyms**: These are combinations of alphanumeric characters that act as surrogates for a name in that they are labels but not in the form of a scientific or common name. Acronyms may or may not include numbers, may be simple or complex. They account for slightly more than 0.1% of the GenBank content with 980 name-strings classified as ‘acronym’ and a further 450 as ‘GenBank acronym’. Approximately 2% of the acronym entries in GenBank are incorrectly classified scientific names (e.g. **Aleiantus incertus Lebis, 1953**). Many acronyms in GenBank are not classified as such, but occur elsewhere such as within ‘scientific names’ and ‘type material’. In some cases, an acronym is associated with a scientific name (see below). Many acronyms end with a ‘V’ and refer to viruses (**RTBV** refers to ‘rice tungro bacilliform virus’), and are reclassified by us as viruses. Some virus acronyms are classified by GenBank as scientific names. Name-strings classified as acronyms occasionally identify a source and the acronym appears unique to that source such that the acronym may be dereferenceable to a taxonomic entity, even if it is not conventionally named. In the last example below, **MBIC** refers to the Marine Biotechnology Institute Company of Japan.

- **RTBV**
- **Bcep781**
- **(Hu/NV/Alphatron/98-2/1998/NET)**
- **Lamprosphaerus sp. JGZ-2004-1**
- **unidentified diatom MBIC10102**

**Anamorph**: Anamorph and teleomorph names are different scientific names used for fungi in the asexual, haploid (anamorph) or sexual, diploid (teleomorph) phase of their growth cycle. They are scientific names. There are 347 anamorph names and 194 teleomorph names (together less than 0.1 % of the GenBank names); many of these will be synonyms.

- **Phaeophleophleospora epicoccoides**
- **Candida guilliermondii var. membranifaciens**

**Authority**: Scientific names which include the name(s) of the author(s) of the name make up about 13% of the name-strings in GenBank. Most of the 250,000 or so ‘Authority’ entries in GenBank duplicate scientific names without author information. The second example illustrates one of the irregular forms of name-strings.

- **Helicobacter pylori (Marshall et al. 1985) Goodwin et al. 1989**
- "**not ""Brucella ovis"" van Drimmelen 1953**"

**Blast name**: This small class of several hundred names identifies taxonomic nodes to help users better understand the taxonomic content of a record. Most are familiar common names, but (erroneously?) includes a number of scientific names as illustrated by the third example.

- **mites & ticks**
- **sea cucumbers**
- **Pseudocosmospora eutypellae C. Herrera & P. Chaverri, 2013**

**Common name**: A vernacular or colloquial name in a natural language. GenBank contains slightly more than 14,000 common names (0.7% of GenBank name-strings) that may identify a species, a higher taxon, or a group name that may refer to several species - such as **Baboon** - which is then disambiguated with a ‘unique name’ (see below). As illustrated, some scientific names are incorrectly included in this class. Some common names use terms that are derived from scientific names and may be spelled identically to the scientific name (**amphioxus**, **eubacteria**). The last example below illustrates a spelling error.

- **Martens's spike moss**
- **Pseudallescheria africana**
- **Mucor miehei**
- **malaria parasite P. falciparum**
- **loosestrife family**
- **baboon**
- **Argentine red shrim**

**Equivalent name**: Alternative names for a taxon which do not satisfy the nomenclatural requirements of synonymy, usually because the name is not code-compliant; many are common or informal names (Federhen 2012). GenBank includes about 21,000 equivalent names, about 1.1% of the GenBank name-strings.

- **Lactobacillus delbrueckii subsp. bulgaricus str. 2038**
- **uncultured cortinariaceous ectomycorrhiza**

**GenBank acronym**: Making up less than 0.1% of GenBank name-strings, these are given priority among acronyms for display purposes when more than one name-string is in use as an acronym for the same entity. The inclusion of more than one acronym is a strategy that ensures that all acronyms are retained for search and indexing purposes. Most are V-acronyms and are treated in this study as names of viruses. A few scientific names are incorrectly included in this class.

- **ThV**
- **nt-1**

**GenBank anamorph**: The 130 anamorph name-strings marked ‘GenBank anamorph’ are given priority for display purposes when more than one anamorph name-string is in use for the same entity. An anamorph name refers to one stage in the life-history of certain fungi (see Anamorph above).

- **Didymostilbe sundara**
- **Tasmanogobius lasti Hoese, 1991**
- **Microsporum canis ATCC 36299**
- **Candida guilliermondii**

**GenBank common name**: Common names marked ‘GenBank’ are given priority for display purposes, and are assigned only if two different common names are in use for the same species. It is a strategy that ensures that all name-strings are retained for search and indexing purposes. There are 25,844 name-strings in this class (about 1.4% of the GenBank name-strings).

- **Lyme disease spirochete**
- **monocotyledons**

**GenBank synonym**: The 2,646 synonyms marked ‘GenBank’ are given priority for display purposes, and are assigned only if more than one synonymic latinized scientific names are in use for the same species. It is a strategy that ensures that all name-strings are retained for search and indexing purposes.

- **Enteromorpha prolifera**
- [**Clostridium] ramosum**

**Includes**: Federhen (Federhen 2012) states that these: “are for names which are useful as retrieval terms but which do not correspond with unique taxa in our classification (e.g. Reptilia)”. They are over 22,000 name-strings in this class (1.2% of GenBank name-strings), many of which include a scientific name and an acronym or strain identifier.

- **Actinobacillus sp. CCUG15571**
- **Achromobacter georgiopolitanum**
- **Characiformes sp. BOLD:AAC1024**
- **Amblyraja cf. taaf INIDEP-T 0140**
- **Pasteurella haemolytica-like sp. (strain 5943B)**

**In-part**: Federhen (2012) states that these “are for names which are useful as retrieval terms but which do not correspond with unique taxa in our classification”. There are 438 instances of this name-class. Many combine a scientific name and an acronym or strain number.

- **zitter rats**
- **Pyrenomycetes**
- **Crassostrea virginica symbiont**
- **Zoogloea sp. strain DhA-35**

**Misnomer**: An incorrect form of a name (Federhen 2012), GenBank includes about 1,300 of these.

- "**not ""Campylobacter fetus subsp. fetus"" Smibert 1974**"
- **endosymbiont sp.**
- **Influenza A virus (A/duck/Yangzhou/013/2009(H6N5))**

**Misspelling**: Incorrect forms of names that have appeared in sequence entries or in the literature, but are useful in searches. There are about 25,000 of these (1.3% of GenBank name-strings), and in addition to mis-spellings (**Hyperamoeba dachnya** for *Hyperamoeba dachnaya)*, they include a mixture of scientific names, names with acronyms, and strain numbers. Misspellings include a large number of name-strings that relate to prokaryotes of which some lack standing in nomenclature (http://www.bacterio.net/).

- **Aeromonas sp. TH096**
- **Drosophila melangaster**
- **Thermus spec.**
- **Anabaena planktonica**
- **Human Herpesvirus-1**

**Scientific name**: GenBank includes almost 1.3 million name-strings in this class, making up 66.8% of the name-strings. As illustrated below, many do not comply with normal understanding of a scientific name (viz. a name written in latin and compliant with appropriate Code(s) of Nomenclature (= 'Code') or, if outside the scope of the Codes, in a form consistent with a Code). The GenBank class includes species, infraspecies, and higher taxon names; annotated names, negated names, scientific and non-scientific generic or higher taxon names, acronyms or strain numbers. Some examples follow.

- **Bacillus xiaoxiensis Chen et al. 2011**
- **Coptodon aff. rheophila 'Samou**'
- **Neodiprion nr. abietis 040.03**
- **Nemoria sp. Janzen27**
- **Zootermopsis hindgut protist**
- **Star grass white leaf phytoplasma**
- **Diatom endosymbiont ex foraminifera MH-2008**
- **Stejneger's beaked whale gammaherpesvirus**
- **transposable plasmid pSET7is**
- **spotted fever group**
- **wall-less spirochete**
- **honey metagenome**
- **diazotroph WWTP**

In addition to these ‘GenBank name classes’, GenBank also includes a field ‘**GenBank unique name**’ that is used to disambiguate duplicates. Most curatorial intrusions comply with nomenclatural and taxonomic expectations, but not all uniques are disambiguated. In the examples that follow, one or more examples of a unique name is/are given after the duplicated name.

- **Bacteria** and **Bacteria <prokaryote>**
- **Treponemataceae** and **Treponemataceae <Spirochaetaceae>**
- **no culture available** and **no culture available <Anaplasma platys>**
- **Inflabilis barati (sic) Prevot 1938** and **Inflabilis barati (sic) Prevot 1938 <Clostridium baratii>**
- **SAG 11-9 [[Chlamydomonas humicola**]] and **SAG 11-9 [[Chlamydomonas humicola]] <authentic strain>**
- **CBS 101750 [[Eurotium parviverruculosum**]] and **CBS 101750 [[Eurotium parviverruculosum]]<holotype>**
- **algae** and **algae <Xanthophyceae>** vs **algae <Chrysophyceae>**
- **red rice** and **red rice<O. longistaminata>** vs **red rice <O. rufipogon>** vs **red rice <Oryza sativa>**

**Synonym**: GenBank includes almost 200,000 name-strings (10.1% of GenBank name-strings) in this category. The term is not used strictly in the nomenclatural sense because, while the majority of name-strings are scientific names, the list includes many strings that are not Code-compliant.

- **Oedipus lincolni**
- **rat dorsal ganglion neuron x mouse neuroblastoma line N18TG2**
- **Euglossa cyanaspis Moure, 1968**
- **Euglossa cyanaspis**
- **Euglossa cyanapis Moure, 1968**
- **euphorine sp. NM-2007**
- **unidentified bacterium**

**Teleomorph**: See anamorph. There are about 200 name-strings in this class.

- **Apiospora**
- **Spinochlamydosporium variabile**

**Type material**: Name-strings associated with type material. A high proportion (71%) of the 78,000 or so name-strings (4.1% of GenBank name-strings) relate to bacteria - not surprising as the Code of Nomenclature for prokaryotes requires the availability of pure cultures, and sequence information can be obtained from the cultures (Federhen 2014). A further 28% of type sequences relate to fungi (Federhen 2014).

- **851004Holotypus**
- **A. R. Smith & al. 1572 (UC)**
- **ATCC 10507**
- **CBS 123208 [[Diaporthe theicola**]]

**Unpublished names**: In addition to the ‘visible’ classes listed above, Federhen (2012) refers to ‘unpublished names’ which are name-strings that are not made public because they may, for example, be pre-publication names. An example given is of **Parapercis lutevittatus** which was eventually published as **Parapercis lutevittata**, but the content was first made visible with the informal surrogate name **Parapercis sp. TYC-2010** until the publication of the final name.

GenBank included 9,146 duplicates relating to 2,335 unique name-strings, the most common duplicated strings were: **environmental samples** (3990), **no culture available** (37), **algae** (21), **Algerian barb** (13), **tsetse fly** (13), **mycorrhizal samples** (11), **Pyrenomycetes** (11), **strain S1** (10) and **Rhodotorula** (10).

**Figure 3.**
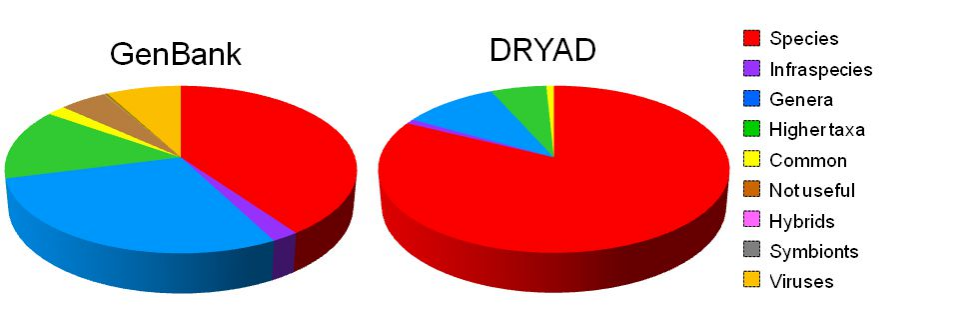
Revised profile of composition of unique name-strings in GenBank and DRYAD (DRYAD name-strings have been pre-processed).

We reclassified GenBank content (Fig. 3, Table 1) after canonicalization and removal of duplicates. Scientific names were mostly derived as the sum of the appropriately classified records in the GenBank classes ‘scientific name’, ‘authority’, ‘anamorph’, ‘teleomorph’, ‘synonyms, ‘includes’, ‘misspelling’, and ‘in-part’; and 590 name-strings from ‘misnomer’ which included canonical elements of genera or species, but excluding negated names and virus names. This reclassification is addressed in more detail below.

The application of ‘Global Names Biodiversity Parser’ to the contents of GenBank produced to 492,154 unique canonicals. The number of name-strings with the same canonical form varied from 1 to 9,742 (**Bacillus).** GenBank has 12,034 name-strings that include the string **Bacillus**; those without a species name will yield ‘**Bacillus**’ as the canonical form. They include:

- **Bacillus sp. PUE-MAN5**
- **Bacillus sp. MJ510**
- **Bacillus enrichment culture clone**
- **Bacillus of abortion**

but not

- **Flectobacillus**
- **Bacillus thuringiensis Berliner 1915**
- **Bacillus thuringiensi**
- **Bacillus fluorescens liquefaciens Flugge 1886.**
- **Jeotgalibacillus**
- **Mageeibacillus indolicus**
- **Columbicola bacillus**
- **Cilia-associated respiratory bacillus**

This approach led us to estimate the number of name-strings that relate to species as just over 800,000. 375,549 unique canonical name-strings were derived from them. This gives somewhat less than the number of species indicated by GenBank (Federhen 2014​). With 22,867 unique canonical infraspecies name-strings, we estimate the number of unique terminal taxa with scientific names in Genbank as about 400,000. Some of these are names of junior synonyms and some are lexical variants of the same name.

### DRYAD content

The nature of the names content of DRYAD differs from that of GenBank. The sample included 17,152 name-strings, reducing to about 13,500 after duplicates were removed. Pre-processing and cononicalization reduced the number of unique entries still further (see below) .

There are differences in taxonomic scope when GenBank and DRYAD are compared. There are few (6) viruses in the DRYAD sample and very few bacteria; and the proportion of name-strings that are scientific names of terminal taxa is higher at about 83% (Table 2).

Presumably reflecting the absence of curation, many original name-strings in DRYAD are not well formed. Extremely few scientific names in DRYAD include authority (although both **Oxalis adenophylla Gillies ex Hook. et Arn.** and **Oxalis adenophylla** are present). Frequent distortions included concatenated names, truncated names, and names without genera. For some, there is a translation table (e.g.  http://datadryad.org/bitstream/handle/10255/dryad.7874/README.txt?sequence=2). Because of the preponderance of concatenated name-strings, the DRYAD content was pre-processed (Table 2) using the interpolated character(s) such as ‘0’, ‘_’, and ‘X’ that are included between generic and species elements to break name-strings into genus and species components and to remove unparseable name-strings. This process reduced the number of name-strings to 7,395, further reducing to 6,948 after duplicates were removed. The concatenated name-strings were sometimes further distorted by being abbreviated to 10 characters.

- **Triticum_aestivum** - the most common form of concatenation with an interpolated underscore (5200 instances)
- **Crassostre** - 10 character abbreviation of *Crossostrea*
- **Ixodidae00** - characters are added to extend the string to 10 characters
- **LissXtimor** - for *Lissoclinum timorense*
- **Danio0reri** and **DanioXreri** for *Danio rerio* (the cyprinid zebra fish)
- **Bombyx0mor** for *Bombyx mori* which is also in DRYAD
- **Gallus0gal** and **GallusXgal** - for *Gallus gallus*
- **HomoXsapie** - for *Homo sapiens*
- **Ptrigonalis** - first letter of genus name plus 9 characters of the species name.

Not all length-adjusted strings are 10 characters long.

- **Dilomasp** - presumably for *Diloma* sp.

Not all name-strings with underscores were associated with a simple concatenation of genus and species elements.

- **Pan__Herm**
- **Didemnum_sp_AB211073.1**
- **Pinoyscincus_jagori_grandis**
- **Unidentified_Enchytraeidae**
- **Homo_sapiens**

Some name-strings include various kinds of annotation.

- **Aporrectodea longa or longa/nocturna**
- **Ipomopsis aggregata and tenuituba**
- **Rhinella ""castaneotica"" s.l.**

The following is an oddity, the significance of **R1** is unexplained, and hence the relationship between the two name-strings is not clear.

- **Oikopleura labradoriensis and Oikopleura labradoriensis R1**

Other examples of issues are given later.

### Cross-mapping

**Figure 4.**
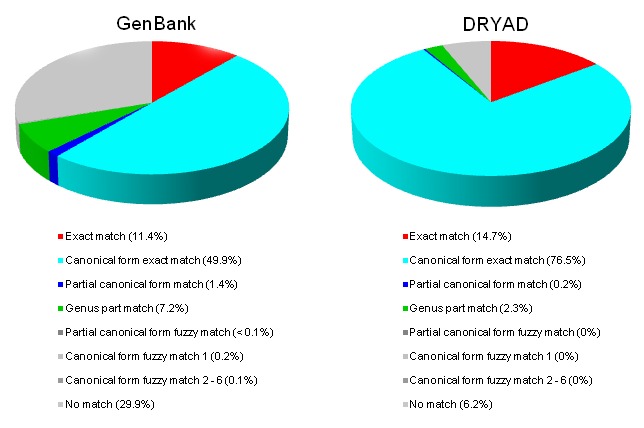
Percentage of unique name-strings from GenBank and DRYAD after pre-processing in each class of match (see text) when cross-mapped to Catalogue of Life. Each name-string was assigned to a single category.

The contents of GenBank and pre-processed and unprocessed (verbatim) DRYAD content were cross-mapped against Catalogue of Life (Fig. 4) and produced 1,988,847 results. The original GenBank name-strings led to 1,974,840 matches - an inflation of about 2.8% because of matches to two or more entries in Catalogue of Life. There were 3,957 unique pre-processed name-strings from DRYAD and these had 6,948 matches, an inflation of about 75% for the same reason. The classes of matches, together with examples are illustrated below.

**1. Exact matches** - significantly, only about 11-15% of the results fall into this class.

- **Balaenidae**
- **Girardinia diversifolia**
- **Bison bison bison**
- **Pseudomonas syringae** but not **Pseudomonas syringae pv. syringae**

**2. Canonical form matches** - canonicalization allows a further 50% (GenBank) to 76% (DRYAD) of the name-strings to be matched. Canonicalization overcomes inaccuracies or inconsistencies of author and date information, intrusions of annotations into name-strings, or duplicates that are created in other ways, such as with different ranking. With canonicalization, the following matched to **Acer cappadocicum var. sinicum Rehd.**:

- **Acer cappadocicum subsp. sinicum**
- **Acer cappadocicum subsp. sinicum (Rehder) Hand.-Mazz.**
- **Acer cappadocicum var. sinicum**
- **Acer cappadocicum var. sinicum Rehder**

With canonicalization, author and/or strain information is removed, with the following matching to **Paludibacter propionicigenes**:

- **Paludibacter propionicigenes**
- **Paludibacter propionicigenes Ueki et al. 2006**
- **Paludibacter propionicigenes CCUG 53888**
- **Paludibacter propionicigenes JCM 13257**
- **Paludibacter propionicigenes str. WB4**
- **Paludibacter propionicigenes WB4**
- **Paludibacter propionicigenes DSM 17365**

Similarly, annotations are eliminated such that the following mapped to **Acanthurus leucosternon.** Annotations such as 'cf' in the first name-string are discussed by Bergstrom (Bergstrom 1988). Different authors us this in different ways, usually to indicate that the taxon in question is NOT *A. leucosternon*, but is similar to it, or alternatively to suggest that the observed individuals represent an unusual form of the species.

- **Acanthurus cf. leucosternon LvH-2007**
- **Acanthurus leucosternon**
- **Acanthurus leucosternon Bennett, 1833**

The following variant spellings all matched to **Indigofera roseo-caerulea**:

- **Indigofera roseo-caerulea**
- **Indigofera roseocaerulea**
- **Indigofera roseocaerulea Baker f**

The following matches would NOT be found if constrained to exact matches of the full name-strings. In these and following examples the name-strings from the sources are given first; the matched name-strings from the target follow.

- **Osedax sp. yellow-patch** matched to **Osedax**
- **Griffonia simplicifolia (M.Vahl ex DC.) Baill.** matched to **Griffonia simplicifolia (DC.)Baill.**
- **Pratia macrodon** matched to **Pratia macrodon Hook.f.**
- **Stagonosporopsis bohemica** matched to **Stagonosporopsis bohemica (KabÃ¡t & BubÃ¡k) Boerema, Gruyter & Noordel. 1997**
- **Lutzomyia (Helcocyrtomyia) hartmanni** matched to **Lutzomyia hartmanni (Fairchild & Hertig, 1957)**
- **Phenylobacterium Lingens et al. 1985 emend. Abraham et al. 2008** matched to **Phenylobacterium**
- **Hyphomicrobium Stutzer and Hartleb 1899 (sic)** matched to **Hyphomicrobium**
- **Brucella abortus** matched to **Brucella abortus (Schmidt 1901) Meyer and Shaw 1920 (Approved Lists 1980).**

Canonicalization is not always beneficial. The following 4 pairs of name-strings with nomenclatural and taxonomic annotations are treated as identical after canonicalization, although this is incorrect. Clearly, there is a need to further refine the matching logic, although the same benefits would arise if taxonomic compilations excluded name-strings that are not code-compliant:

- **Abudefduf saxatilis (Linnaeus, 1758)**
- **Abudefduf saxatilis (non Linnaeus, 1758)**
- **Abudefduf septemfasciatus (Cuvier, 1830)**
- **Abudefduf septemfasciatus (non Cuvier, 1830)**
- **Aegilops triaristata Req. ex Bertol., nom. illeg.**
- **Aegilops triaristata Willd., nom. superfl.**
- **Aegiphila brachiata Schltdl. & Cham., nom. illeg.**
- **Aegiphila brachiata Vell.**

The use of canonicals often produce ambiguous or misleading results with chresonyms, homonyms, concepts, and subtaxa. The following two examples are of positive matches of single canonicalized name-strings of species to homonyms.

- **Asterina gibbosa (Pennant, 1777)** matched to **Asterina gibbosa Gaillard 1897** (a fungus), and to **Asterina gibbosa (Pennant, 1777)** (an echinoderm) and
- **Baileya australis (Grote, 1881)** matched **Baileya australis Rydb.** (a flowering plant) and the moth **Baileya australis Grote, 1881**.

A loss of information associated with canonicalization is illustrated below with examples of concepts, subspecies, or other information.

- **Acacia catechu (L.f.)Willd., Acacia catechu auct. non L., Acacia catechu auct. non Willd., and Acacia catechu (L.f.) Willd.** all had a canonical form exact match to **Acacia catechu (L.f.) Willd.**
- **Cnemidophorus tigris aethiops, Cnemidophorus tigris marmoratus, Cnemidophorus tigris maximus, Cnemidophorus tigris punctilinealis, Cnemidophorus tigris septentrionalis, and Cnemidophorus tigris tigris** all matched (as partial matches) to **Cnemidophorus tigris Baird & Girard, 1852.**
- **Cucumis melo subsp. melo var. conomon** matched (as a partial match) to **Cucumis melo Blanco**
- **Indigofera sp. 'gleichenioides', Indigofera sp. Wilson & Palmer 1776**, and **Indigofera grandiflora B.H.Choi & S.K.Cho** were matched to **Indigofera.**

Canonical matching can also lead to false matches. As with the problems illustrated above with annotated names, the exclusion of non-code-compliant name strings in taxonomic sources would address these errors.

- **Indigofera argentea Burm.f., 1768 non L., 1771** matched inappropriately to **Indigofera argentea L.**, **Indigofera argentea sensu Roxb.**, **Indigofera argentea sensu Baker**, but correctly to **Indigofera argentea Burm.f.**

At a higher taxonomic level, over 40,000 name-strings in GenBank that begin with **Lepidoptera sp. BOLD** had a canonical form exact match to **Lepidoptera**.

**3. Genus part match** are based on the genus component of the name when the remainder of the name-string is absent from the target. This creates taxonomically less precise and therefore inaccurate matches. Some examples are:

- **Yua austro-orientalis** matched to **Yua**
- **Lysandra coridon gennargenti** matched to **Lysandra**
- **Wolbachia endosymbiont of Drosophila innubila** matched to **Wolbachia**
- **Epichloe uncinata** matched to **Epichloë**
- **Pasteurellaceae bacterium 35** matched to **Pasteurellaceae**
- **Bactrocera tyroni species complex** matched to **Bactrocera**
- **Frullania cf. madothecoides Davis 295** matched to **Frullania**
- **Ficus ruginerva** matched to **Ficus du K. Schum. &amp; Lauterb.**
- **Sphagnum** matched to **Sphagnum affine**, **Sphagnum affine Renauld & Cardot**, **Sphagnum aureum**, **Sphagnum aureum C.B.McQueen**, **Sphagnum auriculatum**, **Sphagnum austinii**, **Sphagnum austinii Sull.**, **Sphagnum beringiense**, **Sphagnum beringiense A.J.Shaw, R.E.Andrus & B.Shaw**, **Sphagnum bordasii**, **Sphagnum bordasii Besch.**, **Sphagnum brasiliense**, **Sphagnum brasiliense Warnst.**, **Sphagnum cribrosum**, **Sphagnum cribrosum Lindb.**, **Sphagnum crispum**, **Sphagnum crispum R.E.Andrus**, **Sphagnum cymbifolioides**, **Sphagnum cymbifolioides Muell.Hal.**, **Sphagnum ecuadorense**, **Sphagnum ecuadorense Warnst.**, **Sphagnum inexspectatum**, **Sphagnum inexspectatum Flatberg**, **Sphagnum intermedium (Warnst.) Russow, 1894, non Sphagnum intermedium Hoffm., 1796**, **Sphagnum kenaiense**, **Sphagnum kenaiense R.E.Andrus**, **Sphagnum khasianum**, **Sphagnum khasianum Mitt.**, **Sphagnum leonii**, **Sphagnum leonii H.A.Crum**, **Sphagnum microcarpum**, **Sphagnum microcarpum Warnst.**, **Sphagnum monzonense**, **Sphagnum monzonense Warnst.**, **Sphagnum nemoreum**, **Sphagnum nemoreum Scop.**, **Sphagnum palenae**, **Sphagnum patens**, **Sphagnum patens Brid., 1806, non Besch., 1880**, **Sphagnum perfoliatum**, **Sphagnum perfoliatum L.I. Savicz**, **Sphagnum pulchricoma**, **Sphagnum pulchricoma Muell.Hal.**, **Sphagnum pycnocladum**, **Sphagnum pycnocladum Angstrom**, **Sphagnum sjorsii**, **Sphagnum tenerum**, **Sphagnum tenerum Sull. & Lesq. ex Sull.**, **Sphagnum vitjianum**, **Sphagnum vitjianum Schimp.**, **Sphagnum warnstorfii**, **Sphagnum warnstorfii Russow**, **Sphagnum wheeleri**,**Sphagnum wheeleri Muell.Hal.** (with further canonical matches to **Sphagnum L., 1753**, **Sphagnum sp. Andreas s.n.**, **Sphagnum sp. Andrus 7630**, **Sphagnum sp. BS-2008**, **Sphagnum sp. De Sloover 42750**, **Sphagnum sp. HG-1998**, **Sphagnum sp. Iserentant B-22**, **Sphagnum sp. JL-2005**, **Sphagnum sp. Lafarge Swamp 28-07-02**, **Sphagnum sp. Miehe and Miehe U71-10970**, **Sphagnum sp. Miehe and Miehe U80-11017**, **Sphagnum sp. SB-2000**, **Sphagnum sp. Shaw 10990**, **Sphagnum sp. Shaw 11015**, **Sphagnum sp. Shaw 11195**, **Sphagnum sp. Shaw 11215**, **Sphagnum sp. Shaw 11235**, **Sphagnum sp. Shaw 11267**, **Sphagnum sp. Shaw 11313**, **Sphagnum sp. Shaw 11365**, **Sphagnum sp. Shaw 11390**, **Sphagnum sp. Shaw 11468**, **Sphagnum sp. Shaw 12744**, **Sphagnum sp. Shaw 13169**, **Sphagnum sp. Shaw 13181**, **Sphagnum sp. Shaw 13192**, **Sphagnum sp. Shaw 9680** and **Sphagnum sp. Whitney 992**).

**4. Partial canonical form matches** rely on canonical versions of names and occur if there is a match of, say only of the species element of a infraspecific name. In the case of *Ablepharus*, a skink from Mauritius, one name matched 15 different names in Catalogue of Life with the same canonical genus-species components. Interestingly, none of the 15 names in Catalogue of Life mention Julien Desjardins who established the basionym *boutonii* (Desjardins 1831), but the basionym is present in CoL as **Scincus boutonii Des Jardins, 1831** and treated as a synonym of **Cryptoblepharus boutonii (Des Jardin, 1831)**.

- **Ablepharus boutonii africanus** matched to **Ablepharus boutonii Boettger, 1881**, **Ablepharus boutonii Boettger, 1913**, **Ablepharus boutonii Boulenger, 1887**, **Ablepharus boutonii Boulenger, 1898**, A**blepharus boutonii De Jong, 1926**, **Ablepharus boutonii Loveridge, 1934**, **Ablepharus boutonii Mertens, 1930**, **Ablepharus boutonii Mertens, 1931**, **Ablepharus boutonii Mertens, 1958**, **Ablepharus boutonii Roux, 1910**, **Ablepharus boutonii Sternfeld, 1918**, **Ablepharus boutonii Storr, 1961**, **Ablepharus boutonii Strauch, 1868**, **Ablepharus boutonii Waite, 1929**, and **Ablepharus boutonii Weber, 1890**.

The multiple entries for **Ablepharus boutonii** may be chresonyms, or result from a failure of the relevant GSD (contributor to Catalogue of Life) to include infraspecific elements of a name but include the authorship of the infraspecific element (Flann, pers. comm.)

The following examples illustrate a loss of precision with partial canonical matches:

- **Rattus rattus complex lineage III** matched to **Rattus rattus (Linnaeus, 1758)**
- **Ureaplasma urealyticum serovar 7 str. ATCC 27819** matched to **Ureaplasma urealyticum Shepard et al. 1974 (Approved Lists 1980)**
- **Sargus bipunctatus (Scopoli, 1763) -- valid** matched to **Sargus bipunctatus Costa, 1844**

However, the following examples illustrate inappropriate matches, where the cross-mapping software incorrectly interprets hybrid notation or establishes matches to host names when the record relates to a symbiont.

- **Nepenthes narrow-mouthed frog** matched to **Nepenthes**
- **Cyprus processionary caterpillar** matched to **Cyprus**
- **Virginia winged rockcress** matched to **Virginia**
- **Victoria 'Longwood hybrid**' matched to **Victoria Warren, 1897**
- **Victoria Archipelago frog** matched to **Victoria Warren, 1897**
- **Vesicomya chordata gill symbiont** matched to **Vesicomya cordata Boss, 1968**
- **Ceropegia anjanericax Festulolium braunii** matched to **Ceropegia anjanerica Malpure, M.Y.Kamble &amp; S.R.Yadav**
- **Populus maximowizii x Populus trichocarpa** matched to **Populus maximowiczii A. Henry**

**5. Fuzzy matching** seeks to overcome impediments to matching that are caused by errors that may, for example, truncate names, replace or transform characters. The following examples illustrate mis-spellings.

- **Syzggiam samaragense** and **Syzygium samarangense (Blume) Merr. & L.M.Perry**
- **Tsuchiaea wingfieldii** and **Tsuchiyeae wingfieldii**
- **Dactylethra** and **Daktylethra**
- **Dodekapodorhabdus** and **Dodekapoderhabdus**
- **Owenia hilli** and **Owenia hillii**

Fuzzy matching comparisons are based on canonical versions of the names because the component that is most subject to variation is the ‘Author, date’ element - in part because of the inclusion by some data-sources of chresonyms as if they are synonyms. The tolerance can be adjusted to find matches with a single difference between strings, two differences, etc. The most tolerant and correct match involved 6 differences:

- **Lasidioplodia pseudobromae** matched **Lasiodiplodia pseudotheobromae A.J.L. Phillips, A. Alves & Crous 2008**

About 80% of fuzzy matches based on a single error produced correct matches (Fig. 4), and so improve the potential for interoperability. Examples include:

- **Papaver somnifera** matched to **Papaver somniferum L.**
- **Blumea hieraciifolia var. hamiltonii (DC.) C.B.Clarke** matched to **Blumea hieracifolia var. hamiltonii (DC.) C.B.Clarke**
- **Trichophyton mentagrophyte var. interdigitale** matched to **Trichophyton mentagrophytes var. interdigitale (Priestley) Moraes 1950**

Perhaps revealing a weakness in the algorithm, fuzzy matching frequently failed to correctly match name-strings that had the leading character removed, such as:

- **chneumon dorsalis Fabricius, 1798**
- **radescantia brevifolia (Torr.) Rose**
- **uphorbia trichotoma Kunth**
- **olanum sendtnerianum Van Heurck & Muell.Arg.**

Fuzzy matches perform badly if the source-name-string is not a scientific name. About 1,000 of the 14,000 or so unique common names in GenBank were matched to a scientific name. Many were instances where the scientific name and common name are the same (**Vicugna**), or where the first word in the common-name-string matched or nearly matched a genus name. Some examples of matches between common names and scientific names follow with confidence scores..

- **Engelhardt's mushroomtongue salamander** matched to **Engelhardia** (0.5)
- **Engelhardt's mushroomtongue salamander** matched to **Engelhardtia** (0.5)
- **Rafinesque's big-eared bat** matched to **Rafinesquia** (0.5)
- **Rosa hybrid cultivar** matched **Rosa hybrida Schleicher** (0.75)
- **Lander's horseshoe bat** matched to **Leander** (0.5)
- **Lander's horseshoe bat** matched to **Sander** (0.5)
- **Marini's grenadier** matched to **Marina** (0.5)
- **Crosse's shrew** matched to **Crossea** (0.5)
- **Taczanowski's ground-tyrant** matched to T **aczanowskia** (0.5)
- **Ranunculids** matched to **Ranunculus** (0.5) although **ranunculids minus** matched to **Ranunculus mirus** with a confidence score of 0.75.

Fuzzy matching using canonical versions of names revealed additional issues such as multiple variants of authority information, as indicated below.

- **Bothrops taeniata** matched to **Bothrops taeniatus Wagler, 1824** and to **Bothrops taeniatus Kornacker, 1999** both with a confidence score of 0.75.
- **Bacillus graminis Bibi et al. 2011** matched to **Bacillus gracilis Schaum, 1862**, **Bacillus gracilis Burmeister, 1838**, **Bacillus gracilis (Gray, G.R., 1835)**, and to **Bacillus gramineus Bates, 1865**

Fuzzy matching works well if there is only one other name-string that differs by a single character. Yet this is not always the case, giving multiple errors in the following case of the prokaryotic genus *Mumia*.

- **Mumia Lee et al. 2014** matched **Cumia**, **Mimia**, **Mucia**, **Mukia**, **Numia**, and **Rumia**

**Figure 5.**
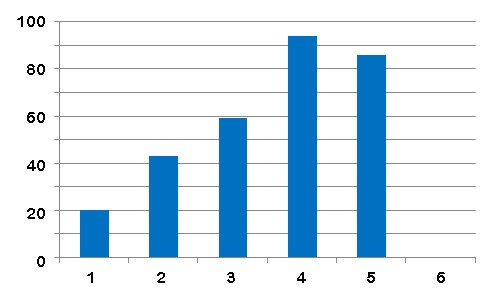
Percentages of 8,097 fuzzy matches that returned questionable results. Horizontal axis indicates the edit distance.

We checked fuzzy matches involving GenBank name-strings by eye to assess how well the algorithm performed (Fig. 5). Matches were regarded as unacceptable if they mapped to more than one target name (the most common issue), or if there was inconsistency with authority, date, rank, and/or subtaxon information in the matching name-strings. As indicated with the examples above, there may be many causes for this - from homonyms, chresonyms (common) or multiple targets with the same degree of difference to the source.

**6. Partial canonical form fuzzy matches** also address errors is in the presentation of the name-string, but taxonomic precision is reduced because the matches are based on only some elements (usually the genus part) of the name.

- **Streptomyces cinnamomeus forma cinnamomeus (sic) Pridham et al. 1956** matched **Streptomyces cinnamoneus**
- **Rosa multifiora var. cathayensis** matched **Rosa multiflora C.P. Thunb. ex A. Murray**

This class included further examples of fuzzy matching errors relating to hybrids and a redirection of emphasis from a symbiont to its host.

- **Pyrus hybrid cultivar** matched **Pyrus hybrida Moench**
- **Phaseoulus vulgaris phytoplasma** matched **Phaseolus vulgaris L.**, and **Phaseolus vulgaris sensu Blanco, non L.**

**7. No Match.** Over a quarter (about 580,000) of the name-strings in GenBank failed to match to Catalogue of Life in any way. Of these about 60% were scientific names, some of which are of relatively familiar organisms (**Porphyra purpurea**, **Emiliania huxleyi (Lohmann) W.W.Hay & H.P.Mohler, Klebsormidium dissectum (F.Gay) Ettl & Gaertner**, and **Prorocentrum micans**). About 14% (74,000) of un-matched name-strings were of genera and about quarter (120,000) were higher taxon names. Non-scientific names that were un-matched included common names (**spirochetes, son-killer infecting Nasonia vitripennis**), symbionts (**Cytauxzoon sp. ex Iberian lynx)**, over 70,000 acronyms (**ATCC 43296**), surrogates (**Psychrophilic bacterium (strain TAE 79)**), negated names (**not Brucella ovis van Drimmelen 1953**), and name-strings that were not useful because they had no biological content (**Organism N 1 Morgan 1906**).

A similar proportion (about 30% or 5,968) of un-pre-processed name-strings from DRYAD also failed to match to Catalogue of Life. Of these, 60% were names of terminal taxa, and a further 15% were names of higher taxa. Again, some were names of familiar organisms (**Plasmodium vivax**), infraspecific taxa (**Zygrhablithus bijugatus crassus**), and negated names (**Lithastrinus cf. moratus**), and some taxonomic areas such as coccolithophorids (e.g. **Zygrhablithus bijugatus** and a mis-spelled variant of that name **Zygrhablithus bijucatus**) were strongly represented among the fails to match. After pre-processing, only about 6% of the name-strings could not be matched to Catalogue of Life.

The high proportion of un-matched names, plus erroneously matched names, represent the scale of the impediment to name-based interoperability of data sets.

### Revised profiles of source content

Based on the insights from the algorithmic approaches and human checks, we further reclassified the content in a fashion appropriate to an agenda of cross-linking data elements based on name-strings (Fig. 3 Table 1). In the following illustrations of various classes, we point to some types of problems that each class presents.

**1. Clade identifiable**

Name-strings that included elements that could be identified to a clade were divided into four subclasses: infraspecies, species, genera and higher. The classes with greatest value when cross-linking are the terminal taxa - species and infraspecies.

**1a. Infraspecies.** This class includes almost 54,000 unique name-strings from GenBank and 377 from DRYAD. Global Names has recorded the following as infrasubspecific ranks:

**morph.**, **f.**, **f.sp.**, **mut.**, **nat**, **nothosubsp.**, **convar.**, **pseudovar.**, **sect.**, **ser.**, **subvar.**, **subf.**, **race**, **α**, **ββ**, **β**, **γ**, **δ**, **ε**, **φ**, **θ**, **μ**, **a**, **b**, **c**, **d**, **e**, **g**, **k**, **, and *.

In this exercise, we treated all on the first line as infraspecific ranks. Infraspecific name-strings represent 1-2% of the content of the sources.

**1b. Species.** Slightly more than 760,000 unique name-strings from GenBank and almost 6,000 from DRYAD were identified as referring to species. Although the name-strings are unique, these numbers include variant spellings, name-strings with and without author information, with and without annotations.

- **Pseudolucia tamara**
- **Pseudolucia tamara Balint & Johnson, 1995**
- **Thermus thermophilus (ex Oshima and Imahori 1974) Manaia et al. 1995**
- **Oleria onega n. ssp. ME-2007**

**1c. Genera.** Almost 550,000 unique name-strings from GenBank and just over 1,600 from DRYAD were placed in this class. Generic names are identifiable partly as uninomial name-strings and not ending with terminations associated with uninomials of higher ranks (e.g. -idae are recommended by the ’zoological code’ for family names or -aceae for plants, algae and fungi), partly because we are aware of them from Latin binomials, and partly because IRMNG (Rees 2008) includes about 95% of all generic names (http://www.cmar.csiro.au/datacentre/irmng/IRMNG_stats_latest.htm). Generic name-strings may or may not include authors and annotations. Some negated species names (see **Gambierodiscus** below) can be rendered to the name of a genus.

- **Paramecium sp**
- **Gambierodiscus aff toxicus**
- **Angophora BOLD 7117**
- **Myxococcus Thaxter 1892 emend. Lang and Stackebrandt 2009**
- **uncultured Candidatus Xenohaliotis sp.**

**1d. Higher taxa.** More than 260,000 name-strings from GenBank and almost 550 from DRYAD include scientific names above the rank of genus or have a non-scientific name that can be reconciled to a scientific name for a clade.

- **Prochlorotrichaceae Burger-Wiersma et al. 1989**
- **Ascomycete from *Sarracenia purpurea* rhizosphere**
- **Environmental Ascomycete sample 2411**
- **unidentified sea urchin**
- **Rhizobiaceae group**
- **ichneumonid wasp MLB-1992**
- **Sphingomonadaceae bacterium TPD06**
- **EF (Enevold Falsen) group 42 bacteria**
- **Chrysauginae gen. chryBioLep01 sp. Janzen200**
- **fungal contaminant of QPX isolate**
- **uncultured sour cassava starch bacterium J1N4**
- **Aenigmarchaeota archaeon SCGC AAA011-O16**
- **fungal contaminant of QPX isolate**
- **aquatic bacterium STS_R2A_38**
- **Cryptophyta gen. sp. Concarneau_14**
- **ectomycorrhizal root tip (Tylospora) 42_Ny1.E1-14.3**
- **unclassified coccolithophorid CCMP 300**
- **unclassified coccolithophorid CCMP300**

**2. Viruses**

Virus nomenclature does not follow the same patterns as the typological Codes for higher taxa. About 150,000 name-strings in Genbank, and 6 in DRYAD, relate to viruses. Some are latinized names, but the majority are not. The use of terms like virus, viroid, phage, sometimes with host or symptoms; higher taxon name endings such as -viridae, -virinae and many acronyms that include ‘v’ relate to viruses and allowed them to be classified as viruses. Examples of name-strings treated as viruses follow.

- **Puumala hantavirus**
- **VESV**
- **Marek's disease virus (MDV)**
- **RV-Tuatara**
- **SARS-CoV (Urbani strain)**
- **Coconut tinangaja viroid**
- **Prochlorococcus cyanophage P-GSP1**
- **Novosphingobium phage N-AFCF0707-15**
- **Yersinia pestis bacteriophage phiA1122**
- **Bat coronavirus M.das/Germany/D3.4/2007**
- **unassigned Alphaherpesvirinae**
- **ssRNA virus taxa unassigned by ICTV**
- **eastern equine encephalomyelitis EEE**
- **Bovine viral diarrhea virus 2 C413**
- **Influenza A virsu (A/Singapore/1/1957(H2N2))**
- **Human immunodeficiency virus type 2 D205**

**3. Common names**

Between them, GenBank and DRYAD include over 37,000 common names (GenBank declares about 40,000 name-strings to be common names, but some refer to viruses and members of other classes), only about 260 are from DRYAD. Only 152 of the unique common name-strings from GenBank found a common-name match in Catalogue of Life. **Vicugna** was the sole exact match, where this name is used both as a common name and generic name for the south american relative of alpaca. About 1,000 common names matched less exactly to scientific names, but as noted above many were instances where the scientific name and common name are the same (**Geranium, Vicugna**, and **Boa**), or where the first word in the common-name-string matched (in the case of **Lacerta**, **bacteria** or **Virginia bluebells**) or nearly matched (**Atlantic John dory** matching the curculionid *Atlantis*), a genus name. 

Common names can be placed into a number of subclasses. Some common names identify a species. When these are included within reconciliation structures, they will be reconcilable to a scientific name of a terminal taxon. Examples follow.

- **Grandidier's Madagascar swift**
- **network woggegong**
- **northern bottlenose whale**
- **terrible hairy fly**

Other common names point to broad groups. Some have been added by GenBank curators to identify more extensive clades, but the last 8 examples were given as the identity of the organisms to which associated information relates.

- **Early diverging fungal lineages**
- **DRIP clade**
- **daisy family**
- **falanouc, Malagasy civet, ring-tailed mongoose and others**
- **vouchered mycorrhizae (Thelephoraceae)**
- **Unidentified Lumbricidae**
- **tree**
- **grasses**
- **crab**
- **asp**
- **bird**
- **fish**
- **flea**
- **fungus**

Common names are not immune to problems associated with variant spellings.

- **blow flies**
- **blow flly**
- **blow-fly**
- **blowflies**
- **blowflly**
- **tommy ruff**
- **Tommy rough**

Some common names refer not to the organism, but to diseases or are otherwise descriptive.

- **Lyme disease spirochete**
- **Microbe de l'agalaxie contagieuse Bridre and Donatien 1923**
- **nematode egg-parasitic fungus**
- **Witches'-broom disease of small-fruited acid lime**
- **unidentified white mycelium 1**
- **pea cyst nematode**
- **sticky caecilian**
- **thin bent rods**

Common names may be in any of about 7,000 spoken languages (http://www.ethnologue.com/). The language in use is not specified, and this adds an additional problem in the reconciliation of common language names.

- **cai xin**
- **calabar angwantibo**
- **makawe o raukatauri**
- **pasang bungkus beranak**
- **Peste-des-petits-ruminants**

Some common names overlap with natural language words and phrases, adding challenges in disambiguation.

- **Maltese cross**
- **Mayo**
- **Medics**
- **similar frog**
- **Tasmania**
- **townhall clock**

Occasional common names are concatenated.

- **CaliforniaJackrabbit**

Some common names simply appeal.

- **depressed flour beetle**
- **fairy's barf**
- **John-go-to-bed-at-noon**
- **laboratory rat**
- **The Thing**
- **violet-washed wave**
- **women's tongues**

**4. Symbionts**

About 6,000 name-strings relating to symbionts may include two scientific names that can lead to matches with the wrong name (**Melia azedarach phytoplasma** was matched to **Melia azedarach** whereas the significant element of the name-string is ‘phytoplasma’, or more correctly, the phytoplasma taxon that is associated with this chinaberry tree). We believe that many instances of names of symbionts can be resolved by GN tools imrpoved to incorporate dictionaries of the names of symbionts, and are aware of terms or sequences of terms that reflect associations such as the use of the term ‘symbiont’, host’, ‘parasite’, or similar term; inclusion of ‘of’, ‘ex’ (also used with other meanings in name-strings) or ‘from’, or the use of inverted commas to declare a relationship. That said, there are over 1,800 references to bacterial phytoplasmas, but there is considerable variety as to how the name-strings for them are presented. The last example suggests that the GenBank material comes from two species.

- **host Paramecium tetraurelia 51KMJ**
- **Zootermopsis hindgut protist**
- **Alvinella pompejana symbiont APG1Bstab9**
- **Inanidrilus exumae associated proteobacterium Delta 8**
- **Alviniconcha aff. hessleri gill endosymbiont**
- **Incompatibility symbiont of Nasonia vitripennis**
- **Chlorella symbiont of Hydra viridis**
- **Wolbachia endosymbiont 1 of Acromyrmex octospinosus**
- '**Ipomoea pes-caprae' little leaf phytoplasma**
- **Ipomoea pes-caprae little leaf phytoplasma**×
- **Onion yellows phytoplasma**
- **Phytoplasma sp. onion yellows**
- **Onion yellows phytoplasma str. 'onion yellows**'
- **Phytoplasma sp. LfY5(PE65)-Oaxaca**
- **Lariskella endosymbiont of Curculio morimotoi**
- **Urosporidium parasite of Stictodora lari**
- **Euduboscquella sp. ex Favella markusovszkyi**
- **Trypanosoma sp. from Abramis brama**
- **Isospora sp. ex Talpa europaea**
- **Isospora sp. Talpa europaea**
- **Prunus armeniaca phytoplasm and possibly**
- **Trebouxia (Asterochloris) photobiont L6**
- **Riftia pachyptila trophosome symbiont**
- **strain KNic within Acanthamoeba castellanii**
- **Pocillopora damicornis/Symbiodinium spp. mixed library.**

**5. Hybrids**

As with symbionts, name-strings that relate to hybrids often involve two names. This can lead to incorrect matching of names (**Populus maximowizii x Populus trichocarpa** matches to **Populus maximowiczii A. Henry).** There are some established conventions as to how to indicate hybrids in addition to using the term ‘hybrid’. The most usual form is to include a symbol that looks like a multiplication sign; but a variety of differently encoded characters can look similar (a Latin letter, letters of other alphabets, the Roman numeral for 10, multiplication signs, and others - х, Х, ×) . GenBank name-strings are consistent in regard to the character used, but this is not true of other sources. The same characters can be used in other ways, such as to indicate an un-named species (**Thiobacillus X Parker and Prisk 1953**) or to mark natural hybrids that have been described with a binomial name. Several hundred name-strings relating to hybrids were encountered.

- **Magnolia heptapeta x Magnolia quinquepeta**
- **domestic duck x muscovy duck**
- **x Aranda**
- **x Cuprocyparis leylandii** (natural hybrid of *Cupressus nootkatensis* and *Cupressus macrocarpa* but also referred to as **Cupressocyparis leylandii** - without hybrid sign - and **x Hesperotropsis leylandii**)
- **Hordeum sp. x Triticum sp.**
- **N18TG-2 mouse (A/J) x C6BU-1 rat (Wistar)**
- **Iris brevicaulis x fulva x hexagona**
- **Sidalcea oregana subsp. oregana x Sidalcea asprella**
- **Saccharomyces bayanus x cerevisiae x cf. kudriavzevii**
- **Nepenthes xhookeriana** - a natural hybrid in this genus of pitcher plants
- **Erysimum x oderatum** - may be a typographic error for *Erysimum odoratum*
- **(Populus alba x Populus glandulosa) x Populus tomentosa**
- **tetraploid red crucian carp x blunt snout bream**
- **Gerbera hybridcultivar**
- **Malus x domestica** is a natural hybrid.

**6. Not useful**

About 100,000 name-strings were deemed to be ‘not useful’ because they contained no direct, indirect, or discoverable reference to a taxon. In some cases, the name-string may be associated with other data sources (Barcode of Life Database = BOLD, culture collections) and in the future may, by invoking the content of those sources, be mappable to a clade. At this time, they cannot be mapped based on the information within the name-string. Various classes of ‘not useful’ name-strings were identified.

**6a. Too fuzzy.** Issues relating to fuzzy matching are discussed above. About one in five matches are incorrect when the tolerance is set to a difference of a single character, but this rises to 40% when matches are sought that allow for a difference of two characters. We regard this as unacceptably high and assign all name-strings that were matched at this or greater tolerances to ‘Not useful’. Despite our classification, some of these matches will be good.

**6b. No genus name.** This class of problem was more prevalent in DRYAD content. It is indicative that the data provider knew what taxon was being referred to with an abbreviated or genus-free name; but that clearly presents problems for re-use of data by others.

- **A. niger**
- **Pmactriformis** (genus initial letter concatenated with species epithet)
- **pluricinctus**
- **vancouverensis**
- **Legumen** (for **Stauroneis legumen**)
- **Rh. axei** (for **Rhabditella axei**)
- **Parnas.phoebus.8**
- **cyrtoloma** and mis-spelled as **cyrt0loma**
- **A affinis**
- **caudata**
- **E. coli**
- **E. caballus**

**6c. Negated names.** A class of about 750 unique name-strings in GenBank do not include reference to a taxon, or if they do, the taxon in question is negated. Typical annotations that negate a name are ‘not’, ‘non’ and ‘nec’; others include ‘cf.’, ‘nr.’ (which indicate the taxon that was studied is similar to but not the same as the one mentioned), ‘aff’ is a firmer indication that indicates that the taxon studied is NOT the one that is referred to. ‘Ab.’ is a taxonomic judgement that the taxon in question is merely a variant (aberration) rather than a taxon in its own right. ‘Scientific names’ that are negated with annotations present problems to canonicalization and parsing. **Streptostele cf. elgonensis 'Nabugabo**' was matched to **Streptostele elgonensis**. Certainly some of these issues can be addressed by improved business logic, and in many cases the identity of an including taxon can be confirmed even if the target species is not - **Campylobacter jejuni-like bacterium** tells us the taxon is a *Campylobacter*.

- **Diphtherophora cf. lata 9 Mile 1-28 LP2-03**
- **Dendrobates aff. azureus CFBH 4203**
- **Anomaloglossus sp. aff. degranvillei**
- **unclassified Circovirus**
- **Labiotermes nr labralis**
- **Labiotermes cf. labralis**
- **Limax cf. graecus sensu Wiktor, 2001 BNM 062845**
- **Russula xerampelina-like sp.**
- **this_is_not_bacteriophage_SfVI**
- **not Bacteria Haeckel 1894**
- **not Thiobacteria Cavalier-Smith 1998**
- **non Lupinus argenteus J.Agardh, nom. illeg.**

Even annotations may be mis-spelled.

- **Scrippsiella aft. hangoei**

**6d. Numbered names** are name-strings that begin with numbers and so fall outside the conventions of scientific names.

- **936**
- **1-Mar**
- **454594**
- **2Helicopsyche murrumba Mosely, 1953** (presumably a typographical error)
- **24-pointed ladybird beetle**
- **44AHJD-like phages**
- **2,4-D degrading transconjugant WD2**
- **16SrII (Peanut WB group)** (relates to phytoplasma, but this is not evident)
- **16SrIX (Pigeon pea witches'-broom group)** (relates to phytoplasma, but this is not evident with the name-string)

**6e. Environmental.** Five thousand or so name-strings refer to a location, habitat, or material that was sampled, rather than to organisms. They contain no information that would lead to one or more terminal taxa. We did not assign to this class those name-strings that refer to a taxon that was encountered from a sample of the natural world (environment) - such as **angiosperm environmental sample**, **Acanthamoeba environmental sample**, or **Thaumatomastigidae environmental sample.**

- **environmental samples** (with over 4,000 instances, this was the most frequently used name-string)
- **Banisveld landfill bacteria ensemble**
- **coal metagenome**
- **tomb wall metagenome**
- **unidentified soil organism R6-122**
- **environmental clone CC-9**
- **Phytodetritus**

**6f. Concatenated names**: Generic and species elements of a name may be concatenated with or without interpolated characters (such as X, 0, _). The most common was to interpolate an underscore character (in about 5,000 name-strings, mostly from DRYAD).

- **corbulasulcata** and **Corbulacotuhensis** for *Corbula sulcata* and *Corbula cotuhensis*
- **ecoli** for *Escherichia coli*
- **Amphiglossus_sp_robustus**
- **Myxine_glutinosa**

**6g. Abbreviated and idiosyncratic names.** Abbreviated names were more common in DRYAD content. There is an overlap with the class where generic names are not included. Many name-strings, especially concatenated names, are limited to 10 characters suggestive of a constraint in the data logging environment. As indicated earlier, some sources offer a supplementary file that translates the terms into taxa. Some abbreviations, such as **Aa** for *Anguilla anguilla*, match (homonymous) genus names.

- **Crassostre**
- **LissXtimor** for *Lissoclinum timorense*
- **Bombyx0mor** for *Bombyx mori*
- **HomoXsapie**
- **P.potto_JCKerbis2889**
- **Cystodytes tam**
- **iki**
- **A. sp. ""Santa Maria"" 2**
- **con**
- **pot**
- **Nbrevis**
- **Bulk ab993**
- **ConBulk H**
- **Cretaceous**
- **Cyan stellers** which may be an abbreviation for *Cyanocitta stelleri*, Steller’s jay
- **Gy910cf Ldmac Ohrid Mace**
- **Burkho cenocep**, the source, DRYAD, also included the full name **Burkholderia cenocepacia**
- **caestogerardi**, again, the source, DRYAD, included the full name **Caestocorbula gerardi**

**6h. Surrogate strains.** More than 17,000 name-strings include reference to strains. Some of these are associated with taxonomic names and were classified into the appropriate clade-identifiable taxon. About 9,400 lacked any taxonomic information and were classified as ‘not useful’. Some include reference to a recognizable data-source (below, UTEX and ATCC) and we presume they can be linked to additional data through that unique source:strain identifier. Some examples follow.

- **strain serial (Mueller et al.) n 189**
- **strain number 81 Dorey**
- **strain Burgdorfer 3-7-female 6**
- **not strain ID03-0748**
- **strain Royal Perth Hospital 13487**
- **strain Twist-Marseille**
- **type strain 130333**
- **strain=UTEX LB 1032**

**6i. Acronyms** are commonly used as or within name-strings. Some of the acronym-containing name-strings contain taxonomic information, but over 72,000 unique name-strings from GenBank lack such information. As with name-strings with strain identifiers, some include reference to a recognizable data-source (below, ATCC) and we presume they can be linked to additional data through that unique source:strain identifier. Some ‘acronyms’ are likely to be abbreviations of a scientific name. Many acronyms ending in V were treated as viruses.

- **acroBioLep01 BioLep01**
- **ATCC 33224**
- **NBRC 14945**
- **309_Lg_sofi_MtRi_Bulg**
- **CDC Enteric Group 11**
- **C 27 Group Ferguson and Henderson 1947**
- **CTEARO**
- **ArceNPV**
- **TMMMV**
- **unclassified SAR116 cluster**

**6j. Organelle.** A small number of entries relate to components of cells, and the containing organism may or may not be identified.

- **Syrian hamster intracisternal A-particle SHIAP18**
- **Intracisternal A-type particle IAP**
- **nucleomorph Pyrenomonas salina**
- **plastid Porphyridium aerugineum**

**6k. Plasmid.** About 600 name-strings included the term ‘plasmid’.

- **Agrobacterium tumefaciens (TI PLASMID PTI15955)**
- **TOL plasmid**
- **yeast plasmid pGKl2**
- **promiscuous plasmids**

**6l. Molecular**: some name-strings from GenBank provide some explanatory molecular context, but without adding a taxonomic identifier.

- **Boolean Integrase Logic XNOR gate**
- **Betasatellites**
- **beta satellites**
- **artificial sequence**
- **Cherry chlorotic rusty spot associated small satellite-like dsRNA A**
- **Plasposon pTnMod-Cm'OTc**
- **Adenoviral expression vector Ad-hiNOS**
- **DGU.US homologous recombination reporter construct T-DNA**
- **unclassified Double-stranded satellite RNAs**

### Overlaps among sources

​​Our primary goal was to determine the level of overlap among the different compilations of names. Starting with the ‘hits’ table, we determined the number of matches for terminal taxa (species and infraspecies - based on the revised classification of original name-strings) among data sources. Prior to the comparisons, all duplicated name-strings and duplicate canonicals were removed to eliminate duplications in sources, chresonyms, and supernumerary hits. Only matches for terminal taxa were included. We did this using both the original (verbatim) suite of names extracted from DRYAD, and again after those names had been pre-processed. The results are shown in Table 3.

With reclassification, GenBank contained unique canonical name-strings of almost 400,000 (398,740) species and infraspecies of which about 82% could be matched to name-strings in Catalogue of Life. After elimination of known synonyms, 257,702 species name-strings and 20,566 infraspecies matched entries in Catalogue of Life. These represented 13.5% and 1.1%, respectively, of the original name-strings in GenBank and 52.4% and 4.2% of all of the unique canonicalized name-strings.

Of the 5,597 unique canonical names of terminal taxa from the original download of name-strings from DRYAD, 31% of the name-strings matched to entries in Catalogue of Life were species, and 2.8% were infraspecific name-strings. After pre-processing, 92.1% of terminal taxa identified as species found a match in Catalogue of Life. Only 5.8% were un-matched. This contrasts sharply with the values of 66% and 73.5% of the verbatim terminal name-strings that could not be matched to Catalogue of Life or GenBank. ALL name-strings of terminal taxa in DRYAD found a match in GenBank after they were pre-processed.

Only 1,905 unique canonical name-strings were common to GenBank, Catalogue of Life and the processed DRYAD name-strings.

### Other challenges with name-strings.

In addition to the issues identified above, we encountered a number of problems that could be addressed with improved discipline regarding conventions of using names by data sources, and by applying a library of appropriate business rules. The various examples above have been chosen to show typical issues and atypical (more challenging) issues (such as **8**).

**1. Marks**: In addition to the use of characters as linkers between concatenated genus and species elements of names, other additions may be made. The role of annotations is not consistent (Bergstrom 1988). As an example, inverted commas are used in a variety of ways: to depict informal names; to indicate varieties or other infraspecific taxa; to indicate host of a parasite; and with prokaryotes to indicate that the generic vehicle, species epithet, or binomial does not meet the requirements of the appropriate Nomenclatural Code. Knowing which applies is relevant to deciding if the name-string should or should not be used to index the associated content. Some annotations have been added by GenBank to disambiguate otherwise indistinguishable name-strings.

- !**Helicteres baruensis Jacq.**
- '""**Scopulibacillus"" Lee and Lee 2009**
- "**Pseudomonas mangiferaeindicae' Patel et al. 1948**
- ?**Lobivia leptacantha Rausch** This may be a statement about uncertain identification
- '**Pseudophoenix sargentii' yellowing phytoplasma**
- [**Bacillus] thermocloacae** - the hard / square brackets parentheses make a taxonomic statement as to the uncertain status of the taxon
- [**Actinomadura] sp. ATCC 39727**
- ]**Haemophilus] felis** (rare example of an incorrect hard bracket combination may have arisen as a typographic error)
- **(Hu/SV/Park/1994/US)**
- **Aptostichus 'schlingeri**' - inverted commas indicate non-code-compliance
- '**Geophagus' steindachneri** - inverted commas indicate non-code-compliance
- '**Formosa crassostrea**' - inverted commas indicate non-code-compliance
- '**Phlomobacter**' - inverted commas indicate non-code-compliance
- **Pyramimonas sp. 'Greenland**' - inverted commas indicate a subtaxon
- **Paramecium sp. 'UK 03**'- - inverted commas indicate a subtaxon
- **Hyperolius viridiflavus subsp. 'ngorongoro**' - inverted commas indicate a subtaxon
- **Antirrhinum sp. 'Floral Carpet Mix**' - inverted commas indicate a variety
- '**Picris echioides' phyllody phytoplasma** - inverted commas indicate a host
- '**DiaphantaX' chryseres** - *Diaphanta chryseres* (Turner 1898) is a moth, the significance of the X, which is encountered with other generic names is not clear.
- **CBS 101750 [[Eurotium parviverruculosum]]<holotype>**
- **pigweed <Chenopodium rubrum>**
- **pigweed <Chenopodium album>**
- **red rice <O. longistaminata>**
- **red rice <Oryza sativa>**
- **Platycladus orientalis cv 'Flagelliformis**' (**Platycladus orientalis flagelliformis** is also listed by GenBank)

**2. Strings with characters missing, characters added, mis-spelled, or abbreviated.** Some of these may be addressable by fuzzy matching, but as that can also lead to errors, it would be desirable to be able to annotate these name-strings and link them to the correctly spelled name.

- **chneumon dorsalis Fabricius, 1798**
- **fiPineus pini (Gmelin, 1789)**
- **olanum sendtnerianum Van Heurck & Muell.Arg.**
- **Oxy. cachemiriana**
- **RRorippa hybosperma (O.E.Schulz) Jonsell**
- **sammodictyon panduriforme (W.Gregory) D.G.Mann**
- **Lilium_regale**
- **Hypericum patulum_**
- **Anolis_ernestwilliamsi**
- **Prochlorococcus_marinus_subsp_marinus**
- **Candidatus_Portiera_aleyrodidarum**
- **CRYPTOMERIA_JAPONICA)**
- **Anthaenantia villosa** (two spaces between genus and species elements)

**3. Capitalization issues**: Some of the software relies on the capital at the start of a genus name to identify latin names, and so unusual uses of capitalization may be a source of problems.

- **litoria ewingii and litoria moorei** (Australia’s whistling tree and motorbike frogs) lack capitalization and are missed by GN tools
- **concentricavalva** - a non-capitalized genus name (it’s a fossil clam) could be mistaken for a species or for a concatenated binomial.
- **ursirivus** it is not clear if this is a species name without a genus, or a genus without a capital; other entries make it clear that it is a genus without a capital (**Ursirivus pyriformis)**
- **ARABIDOPSIS THALIANA** and **SECALE_CEREALE** - all capital characters
- **TAMARIX_androssowii**, an unusual mix of capital and lower case letters
- **Staphlococcus Aureus**, species epithet with a capital, risking its interpretation as a subgenus or an author.

**4. Annotated** in various ways; often with taxonomic notes or with conventions that have meaning within certain clade (such as ‘candidatus’ to indicate bacterial names that are not yet fully compliant with the code - see below).

- **Bielzia Schur, 1866, nom. rej.**
- **Calothysanis auct. nec Huebner, 1823**
- **Candidatus Allobeggiatoa halophila Hinck et al. 2011**
- '**Chitinophaga terrae' An et al. 2007**
- **elevated to species Nasuella meridensis Helgen et al. 2009**
- **Sphaerisporangium corrig. Ara and Kudo 2007 emend. Mingma et al. 2014**
- **this was Neospora bovis**
- **Marionina communis; Enchytraeidae**
- **Valid as Hydrolagus lusitanicus Moura, Figueiredo, Bordalo-Machado, Almeida & Gordo, 2005**

**5. Candidatus.** The rules of nomenclature for bacteria are challenging and require, among other things, the availability of a culture of the taxon in question. Much of the natural richness has never been cultured (Stewart 2012, Tandogan et al. 2014), and presents an excessive cost in time to achieve. Consequently, conventions have appeared to allow names to be presented even when they are not fully compliant with the relevant code. One solution is to refer to the taxon as ’candidatus’ (Murray and Stackebrandt 1995); another is to use inverted commas to indicate the name is not yet code-compliant:

- **Candidatus Phytoplasma spartii Marcone et al. 2004**
- '**Candidatus Phlomobacter**'

**6. Chresonyms** are formed when scientific names are coupled with the names and dates of authors who are not the authors of the basionym nor combination but who referred to the organism (Smith and Smith 1972). That is, the name-string does not deal with a nomenclatural act and is not code-compliant. Rather, these 'name+author' combinations indicate a usage of a name or a concept for a name. Other inappropriate name+author combinations (here referred to as apparent chresonyms) arise if a name element (such as subspecies) are excluded but the author of the element is retained. We refer to these as apparent chresonyms. In some taxonomic areas, chresonyms are incorrectly included within synonymy lists. Of almost 1,400,000 matches between GenBank and Catalogue of Life, 98,000 involved matches to two or more names in Catalogue of Life, involving 43,000 unique name-strings. These result from matches to homonyms, chresonyms or apparent chresonyms. Chresonyms present disambiguation problems and, if not attended to, give an inflated impression of the number of species in a list. As an example, the species name *Naja haje*, the Egyptian cobra, described as *Coluber haje* by Linnaeus, matched to:

- **Naja haje Mertens, 1937**
- **Naja haje (Linnaeus, 1758)**
- **Naja haje Boettger, 1887**
- **Naja haje Peters, 1854**
- **Naja haje Fischer, 1885**
- **Naja haje Scortecci, 1932**
- **Naja haje Bogert, 1943**
- **Naja haje Hallowell, 1857**
- **Naja haje Wallach Et Al., 2009**
- **Naja haje Valverde, 1989**
- **Naja haje Broadley & Howell, 1991**
- **Naja haje Peters, 1873**
- **Naja haje Jan, 1863**

The Catalogue of Life refers to these name-strings as synonyms, which they are not. **Naja haje annulifera**, **Naja haje anchietae**, and **Naja haje arabica Scortecci 1932** also match to the same bundle of name-strings because those subspecies, not included in the Catalogue of Life, are matched based on the canonical species versions of the name-strings.

A second example, also a reptile, is **Cnemidophorus sackii stictogrammus Burger 1950** which matches to:

- **Cnemidophorus sackii (fide Maslin & Secoy, 1986)**
- **Cnemidophorus sackii Alvarez Del Toro, 1982**
- **Cnemidophorus sackii Burger, 1950**
- **Cnemidophorus sackii Davis & Smith, 1952**
- **Cnemidophorus sackii Schmidt & Stuart, 1941**
- **Cnemidophorus sackii Smith & Taylor, 1950**
- **Cnemidophorus sackii Smith, 1946**
- **Cnemidophorus sackii Smith, 1949**
- **Cnemidophorus sackii Wiegmann, 1834**
- **Cnemidophorus sackii Zweifel, 1959**

A third example is offered by the plant **Corchorus aestuans L** (jute, foku) which matches to the following items in Catalogue of Life.

- **Corchorus aestuans L.**
- **Corchorus aestuans Herb. Madr. ex Wall.**
- **Corchorus aestuans Blanco**
- **Corchorus aestuans Forssk**.

Chresonym problems tend to be associated with particular taxonomic areas, suggesting that the problem arises from the conventions used in particular Global Species Databases that contribute to the Catalogue of Life.

**7. Surrogates** are strings that take the place of a name. They may take a variety of forms, such as acronyms, culture or strain numbers, or a stand-in for a clade. In some cases such as the **PS** example below, strings overlap, allowing identification of the taxonomic target. Other surrogates include reference to a source and an acronym that is likely unique in the context of the source, such that the information may be linkable to other data on the same species via the acronym.

- **Acari sp. BOLD:AAH6618**
- **EF (Enevold Falsen) group 42 bacteria**
- **Porcine transmissible gastroenteritis coronavirus strain Miller**
- **strain PS**
- **strain PS [[Dechlorosoma suillum**]]
- **ZSI/WGRC V/A 857**

**8. Parentheses (brackets)** occur in name-strings. In some cases, they codify a particular piece of information. In the first two examples, the author(s) in parentheses were responsible for creating the basionym. The inclusion of the basionym author is useful as the combination of species epithet and basionym author in taxonomically proximate areas may indicate a homotypic synonym - a valuable insight to the processes of reconciliation and resolution.

- **Xanthomonas populi (Ride 1958) Ride and Ride 1978**
- **Najas guadalupensis subsp. floridana (R.R.Haynes & Wentz) R.R.Haynes & Hellq.**

Elsewhere, parentheses are used to indicate a hybrid (in these two examples a hybrid marked by the parentheses is hybridized with another species). It is more common to encounter this format with plant names.

- **(Citrus unshiu x Citrus sinensis) x Citrus reticulata**
- **(Anopheles sinensis x Anopheles kleini) x Anopheles sinensis**

Square parentheses are used with prokaryotes as one of several ways to indicate informal or uncertain classification or identification.

- [**Bacillus] sp. KITNT-3**
- [**Frankia] sp. HSIi8_AKM4**
- **Myroides [odoratimimus] CIP 103059**
- [**Pasteurella] aerogenes-[Pasteurella] mairii-[Actinobacillus] rossii complex**

Conventional and square parentheses may be used to carry supplementary annotations, such as location or host, or in the context of identifiers of strains.

- **Tomato leaf curl betasatellite-Panipat 1 [India:Panipat:Papaya:2008**]
- **Bean yellow mosaic virus-[Hibiscus rosa-sinensis**]
- **Rotavirus A RVA/pig-wt/JPN/pig9-59d/2003/G11P[27**]
- **Rotavirus A RVA/Human/NCA/125L/2010/G3P[8**]
- **Rotavirus A RVA/Human-wt/ARG/Arg2842/2010/G12P[8**]

In the following examples, double square brackets indicate the taxon referred to using an identifier from some organization (IMI relates to Kew Garden in UK, CBS to the Fungal Biodiversity Centre in Netherlands, ATCC to the American Type Culture Collection). Double parentheses are used to indicate type material in GenBank.

- **IMI 278373 [[Aspergillus glaber]]**
- **CBS 492.91 [[Chrysosporium botryoides]]**
- **CBS 491.91 [[Chrysosporium pyriforme]]**
- **[[Arthrographis alba]] CBS 370.92**
- **ATCC 35419 [[Bacteroides suis]]**

Parentheses are sometimes used to indicate a synonym - in this case, two name-strings are used to refer to the same aphid.

- **Buchnera aphidicola strain 5A (Acyrthosiphon pisum)**

Other uses of square brackets, for example in the context of authorities for names. are less clear, especially when the latter example of the Himalayan Sergeant was described by Kollar as *Athymna opalina* in 1848 NOT 1844. A further complication in this case is that the generic name is a homonym, and the correct name is *Parathyma opalina* (LepIndex record for Athyma opalina).

- **Glaucostegus thouin (Anonymous [Lacepede], 1798)**
- **Athyma opalina (Kollar, [1844])**

**9. Repetitive entries** occur, but are rare.

- **Boreophyllum birdiae Boreophyllum birdiae (Neefus et A. C. Mathieson) Neefus**

**10. Inconsistent encoding** of characters does present a few problems. Latin1, UTF-8 and UTF-16 are most popular encodings used in biodiversity studies. If the name of the author (usually) or the name-string (rarely) includes characters outside of the 128 bits of ASCII code, names converted from one encoding to another incorrectly will have problems. Some examples are:

- **Bembidion concolor BrullÃ©, 1839**
- **Lacerta vivipara FejervÃ¡ryi, 1923**
- **Lacerta vivipara Mayer, BÃ¶hme, Tiedemann & Bischoff, 2000**

**11. Other challenging name-strings.** The following indicate some of the entries as name-strings that cannot be linked to other content:

- ]**commercial sponge**
- **anaerobic hyperthermophile Gorda1**
- **coast**
- **Cretaceous**
- **ctenbiolep01 biolep09**
- **E**
- **E.W.A. Boehm, G.K. Mugambi, S.M. Huhndorf & C.L. Schoch 2009**
- **endosymbiont sp.**
- **HERBS**
- **Host**
- **pot**
- **ID18-like**
- **inconnu**
- **Kluyver and van Niel 1936 emend. Barker 1956 (Approved Lists 1980), nom. cons., emend. Mah and Kuhn 1984**
- **Leochilus x Macradenia x Oncidium x Rodriguezia**
- **long**
- **miscellaneous nucleic acid**
- **no culture available**
- **not strain ID03-0748**
- **Pan__Herm** (is this *Pan* the genus or Pan an abbreviation?)
- **Phytodetritus**
- **Ranunculids minus**
- **strain X**
- **test organism**
- **unidentified organism**
- **unclassified Group 1 species**
- **unknown organism**
- **unknwon**

## Discussion

some The emergence of an integrated environment for the management of digital biodiversity data requires changes to the political and legal frameworks of research, to sociological practices, an extended funding model that has an emphasis on service rather than discovery, and infrastructural changes (Hardisty and Roberts 2013, Patterson 2014, Thessen and Patterson 2011). This study was conducted in the context of a names-based infrastructure (Patterson et al. 2010) and sought to evaluate how ready we are to interconnect data environments by means of the names, particularly those of terminal taxa (species and infraspecies).

Of the 400,000 unique canonicalized name-strings from GenBank that relate to terminal taxa, 82% could be matched to entries in Catalogue of Life, but only after processing. This confirms the potential and practicality of a name-based cyber-infrastructure to interconnect digital data on biodiversity, and the importance of the use of scientific names as metadata. The level of overlap is consistent with the metric that Catalogue of Life has compiled about 85% of all species names. Similarly high match rates were found between pre-processed name-strings from DRYAD and GenBank (100%) or Catalogue of Life (92.1%).

On the negative side, the level of matching without names-processing tools is poor. Only slightly more than 10% of name-strings in sources have an un-aided exact match with elements in the target (Fig. 2). That is, most of the names-as-compiled are not suited for cross-linking. This is particularly evident for the uncurated names in DRYAD, where pre-processing lifted the match of terminal taxa with Catalogue of Life and GenBank from 31% to 94% and from 25% to 100%, respectively. Terminal taxa account for 25% and 45% of the name-strings in GenBank and DRYAD, respectively. In the case of GenBank, over 1 million unique name-strings cannot be associated with terminal taxa in Catalogue of Life. This large body of un-matched name-strings and name-strings that do not relate to terminal taxa are the primary challenges to the effectiveness of a names-based infrastructure. 

As illustrated in the Results section, aberrations in name-strings that make them un-matchable arise for many different reasons, and no single solution will address them. Yet, if name-strings are in the form of scientific names, then parsing and canonicalization will likely ensure that most can be cross-mapped, with some caveats. More effort can and should be made to ensure that well-formed scientific names are part of the data records. If the name-strings are not in the form of scientific names, then devices are needed to place them in the same organizational framework as scientific names, such as by reconciling them to scientific names. The following discussion relates to issues arising.

### Molecular identifiers

The value of molecular identifiers (Barcodes) for taxa is immense, allowing cost-effective routine collection of occurrence data and evaluation of ecological associations, cryptic species, assessment of diversity as well as enabling phylogenetic and other studies (Hebert et al. 2003, Waugh 2007). The integration of the identifiers as surrogates within a names-based infrastructure is achieved through algorithmic analysis of similarities to create bundles of identifiers that may correspond with species (BINs, Ratnasingham and Hebert 2013), and with the inferred phylogenetic (=taxonomic) location within a global classification scheme being achieved through analyses of molecular similarities (Hinchliff et al. 2015). Hinchcliff and colleagues provide a very extensive tree (dendrogram) that includes mostly the entities for which we have molecular data. In it, a large number of the ‘tips’ are not named species, but are entities labelled with molecular identifiers. It is unlikely that many such entities will be resolved to named species. This is most evident with prokaryotes. Given the exacting standards for code-compliance (i.e. availability in culture), a growing number of entities will either not be given any name, or will have interim names identified as such with the term ‘candidatus’ or other marker. It is urgent that molecular identifiers and names are managed together as alternative tokens for taxa, a point addressed for fungi by Schoch et al. (2014) and for bacteria by Federhen and colleagues (Federhen 2014, Federhen et al. 2016). In order to connect content attached to names to content associated with molecular identifiers, the integration process should include the mapping of molecular identifiers to species. This requires a continuing investment in routine sequencing of voucher material (a sample that is also preserved for further analysis if needed). 

We recommend that name-strings that identify molecular sequences (including BINs) should be included within a names-based cyber-infrastructure. The name-strings should be dereferenceable to the associated sequence data. An array of services will be required to keep BINs up to date, to place the molecular identifiers within taxonomic schemes, and to resolve to names of terminal taxa where possible.

### Common names

GenBank content, when reclassified in this exercise, included over 38,000 unique common names. Excluded from the GenBank total are names of viruses, or names which combine both scientific and common elements (**Haplochromis sp. 'big blue**'). Catalogue of Life has, at the time of writing, about 330,000 common names. Yet, only 152 of the unique common name-strings from GenBank found a common-name match in Catalogue of Life. About 1,000 common names matched to a scientific name, but many were instances where the scientific name and common name are the same (**Geranium, Vicugna**, and **Boa**), or where the first word in the common-name-string matched (in the case of **Lacerta**, **bacteria** or **Virginia bluebells**) or nearly matched (**Atlantic John dory** matching the curculionid *Atlantis*) a genus name. Common names do not have a useful role in interconnecting distributed data on biodiversity, but have value in their familiarity. Common names need to be identifiable as such so that names-management tools do not confuse common names with scientific names. Reconciliation services should include common names so that content labelled with scientific names can be accessed through common-to-scientific name reconciliation. Resolution services need to take account of language, locational and other differences in what a common name refers to and which names are most widely used. The integration of the achievements of common-names projects such as OpenUp! (Berendsohn and Güntsch 2012) with the reconciliation groups of a names-infrastructure is very desirable.

### Scientific names management

**Suitability for reconciliation.** Existing author, editorial, and curation practices when coupled with basic parsing and canonicalization tools have a high level of performance with names of terminal taxa - such that reconciliation and resolution is a feasible way of building a common index for distributed data. An alternative to reconciliation is the use of standardized names compilations as reference systems (Boyle et al. 2013, Zermoglio et al. 2016). Standardized lists promote consistency and provide gold-standard material for the last step in reconciliation - that of name resolution. While such lists may be useful, they are are expensive to maintain, do not address the problems associated with multiple points of view, nor address the management of now obsolete names in older documents, nor taxonomic concepts, nor the dynamic nature of taxonomies and phylogenies (Franz and Thau 2011). Standard lists need to be able to call on reconciliation and resolution to keep pace with name changes and name introductions. 

**Taxonomic precision**: Of the almost 500,000 unique canonicalized scientific name-strings in GenBank, about 100,000 referred to genera or higher taxa. Some of these will have been introduced by GenBank for managerial or navigational purposes. Names that cannot be related to terminal taxa, such as **Carnivora** are not very useful for content management. Similarly, precise names that are taxonomically inaccurate or agnostic, are not useful. Examples such as **Ascomycete from Sarracenia purpurea rhizosphere, Paramecium sp**; or **Gambierodiscus aff toxicus** are only identifiable to non-terminal clades. In some cases, the name-string contains information that may allow greater accuracy to be achieved through a cross-link to external sites - **Angophora BOLD 7117, Sphingomonadaceae bacterium TPD06**, **ATCC 25593 [[Rhodococcus corallinus**]] and **CCAP 276/37 Holtmann 1977-5903 [[Scenedesmus pectinatus var. distendus**]]. Protocols to acquire information from cross-links need to be implemented. In the interim, we recommend that ecologists improve taxonomic skills and preserve voucher specimens for subsequent confirmation of identification.

**Curation.** GenBank content is curated (Federhen 2012, Federhen 2014), but DRYAD is not. As a result, DRYAD has many idiosyncratic representations of name-strings. The most common being concatenation with or without interpolated characters and with or without abbreviation to 10 characters. The concatenations were addressed by pre-processing with regular expressions - with a dramatic improvement in cross-matching to other sources (Table 3). The library of expressions needs to be continuously improved as each new problem is identified. Other solutions include translation tables but such tables need to be included within reconciliation services of a names-based infrastructure. In addition to idiosyncrasies, about 400 name-strings relating to scientific names in DRYAD lacked the genus name (**S hangoei** and **virescens**). About one name-string in 20 in both GenBank and DRYAD contained no taxonomically useful information. As for the future, we see no benefits from the continued use of idiosyncratic versions of name-strings. Manual curation is tedious. Both can be addressed using open on-line name validation tools as part of future-proofing practices (see below).

**Taxonomic scope.** Some taxonomic areas are well represented in some sources but not others; GenBank is predictably rich in information about viruses and prokaryotes (‘predictably’ - because these taxa are mostly discoverable by molecular techniques). Catalogue of Life does not claim comprehensive coverage, and given the reliance on the Global Species Database model, some areas - such as *Melaleuca* (oddly), viruses, algae, other protists, and prokaryotes - are poorly represented. This may account for many of 15% unique canonicalized scientific name-strings from GenBank that do not find a match in Catalogue of Life. Devices are needed to include the missing taxa.

Of 150,00 name-strings referring to viruses only 14 found a match in Catalogue of Life, and 4 of these were matched on the host of bacteriophages. The virus Code (International Committee on Taxonomy of Viruses 2011) differs in character to codes for plants, animals, and prokaryotes. The challenge of managing information about viruses can only be achieved with an open compilation of all names and name-strings that point to viruses and their inclusion with comprehensive lists of names. Given the importance of molecular data in discriminating among types of virus, this task may fall to the compilers of sequence data.

In the case of prokaryotes, some of the relevant content is associated with interim and surrogate names, such as candidatus names, or the use of inverted commas and hard brackets. The need for interim name-strings and surrogates is a result of the stringent requirements in the code for nomenclature of prokaryotes (Parker et al. 2015). Modern sequencing of environmental samples continues to reveal very large numbers of previously undescribed prokaryotes, such that the taxonomic community will be unable to comply with the code for anything more than a tiny minority of the revealed diversity. Surrogates and interim names do and will have high value as pointers to information. They need to be integrated within names-compilations, ideally in a standard form. Again, given the importance of sequence information to discriminate among taxa, this task may also fall to the compilers of sequence data.

Various clades of plants and animals are not well represented in major compilations, but the absence of coverage of algae is particularly notable. This may be because of the extreme stance taken by AlgaeBase in limiting re-use of content (Patterson et al. 2014). This matter is addressed under the discussion of copyright. We hold the view that all data should be made freely available, and ideally linked to tools that will monitor usage and re-usage so that credit can be given to authors and compilers of this information.

**Synonymy / chresonymy / ambiregnal taxa**: 

**Synonyms** are needed to develop reconciliation groups that are the essence of a names-based infrastructure. We do not know how many synonyms (on average) to expect. Catalogue of Life holds about 8 synonyms for every 10 species (but they include chresonyms - see below); FishBase assessed the ratio closer to 30:10, the estimate for fungi is 17 synonymous names for 10 species, a list of Chinese mosses about 1 synonym per species, slime moulds (via eumycetozoa.com) have 20 synonyms for 10 species. Given that the Global Names Index contains about 20 million name-strings (many of which will be dirty, variant spellings, or canonical versions) for about 2 million named taxa, and has been rendered into 7.3 reconciliation groups, we suspect the FishBase estimate is the more accurate indicator. 

Synonyms are either homotypic (the names are based on the same type material and referred to as objective or nomenclatural synonyms), or reflect the view that two nomenclaturally compliant names refer to the same species - that is are heterotypic (=subjective or taxonomic) synonyms) (Remsen 2016). Synonymy lists are rarely complete. Synonym lists vary in quality, containing different spellings, chresonyms, and taxonomic statements such as pro. parte. and sensu auctt. We detected differences of opinion between Catalogue of Life and GenBank as to the best name for a taxon, but because the GenBank name was not included as a synonym in Catalogue of Life, the name-strings and associated content could not be matched. Synonymy lists may include contradictory information - as in the case of **Lacerta capensis Smith, 1838** which is given by Catalogue of Life as a synonym of both *Meroles ctenodactylus* (Smith, 1838) and of *Pedioplanis laticeps* (Smith, 1849), or when spelling variants are included as synonyms as in the case of **Bufo flavolineatus Vellard, 1959** and **Bufo flavilineatus Vellard, 1959** - referred to as synonyms of **Rhinella spinulosa (Wiegmann, 1834)**. Further effort in compiling synonymy lists will improve the interconnectability of distributed content.

**Chresonyms** are formed as a combination of the latinized components of the scientific name together with the author of the paper in which the name is used (Smith and Smith 1972). Such uses do not comply with codes of nomenclature, and so should not be included in synonymy lists. With the use of canonical versions of name-strings, chresonyms inflate the perceived matching of lists. Apparent chresonyms are instances of taxon+author combindations that do not refer to nomenclatural acts, and may, for example, because if subspecific taxa are excluded while the authors for those taxa are retained. As noted above, a junior synonym for the snake eyed skink from Mauritius, **Ablepharus boutonii africanus**, matched 15 variants of *Ablepharus boutonii*. 43,000 unique name-strings matched two or more names in Catalogue of Life. Some of these matches will result from canonical matching of species and subspecies, from errors in data entry, and there are a small number of instances of homonyms which match if canonical forms of names are used. Chresonyms have the potential of confusing reconciliation - as in examples where one name in Catalogue of Life is given as a synonym of two different species (**Lacerta caucasica Engelmann Et Al., 1993** is indicated as a synonym of *Darevskia alpina* (Darevsky, 1967) and *Darevskia daghestanica* (Darevsky, 1967)). Chresonyms should be removed from synonymy lists, a task that could be achieved in the long run by filtering Catalogue of Life content through nomenclatural registries such as Index Fungorum, IPNI, and ZooBank.

**Ambiregnal issues.** More than one set of nomenclatural rules may be applied to some groups of microalgae. They are ‘ambiregnal’. Cyanobacteria may be subject to botanical or bacterial conventions, while euglenids, dinoflagellates, stramenopiles, collar flagellates, cryptophytes, and others have been subject to both botanical and zoological conventions. The result is that two names may quite legitimately be applied to the same taxon (Patterson and Larsen 1991, Patterson and Larsen 1992). The alternative names need to be included within lists of species.

Given the value of synonymy lists in reconciliation, synonymy lists should be complete, include alternative names of ambiregnal taxa, and exclude chresonyms if they are to underpin production-grade (>95% satisfaction) reconciliation services.

**Concepts.** 315 name-strings contained the term ‘sensu’. This indicates that the entry refers to a more precisely defined taxon than is achieved with the name-string alone (Berendsohn, 1995). The same is indicated by the term ‘sec.’, although this term did not occur in this study. Various efforts are underway to manage concepts (Berendsohn and Geoffroy 2007, Craig and Kennedy 2008, Franz and Cardona-Duque 2013, Franz and Peet. 2009, Franz et al. 2015, Lepage et al. 2014, Remsen 2016, the Taxonomic Tree Tool; A logical model for linking taxonomic knowledge using linked data, and TaxonConcept.org ). Most tools establish the existence of similar or different concepts on the basis of the taxonomic tree-path (parents), sister taxa, and subordinate taxa. The approach is sensitive to taxonomic completeness and conventions of sources under comparison. The use of concepts draws attention to finer granularity than can be achieved with names on their own. But, as different concepts with the same name overlap, the unambiguous definition of concepts by reference to defining characteristics will be needed if they are to be used in organizing biodiversity information. Yet, the characteristics that allow overlapping concepts to be distinguished are not codified in a standard way, are rarely specified, and, if accessible, can only be established with examination of taxonomic texts. As concepts are little used and cannot be readily identified, we do not regard the practical challenges of managing concepts as currently tractable on a large scale, and so do not regard this area as ready for inclusion in a cyber-infrastructure.

### GN TOOLS: Evaluation and Future Work

**Canonicalization.** Canonicalization, the removal of spurious elements from name-strings to leave the Latinized elements, is dependent on the GN parser. Of the 1.9 million unique name-strings in GenBank, about 1.61 million are scientific names, of which only 219,216 match to Catalogue of Life. When the scientific names are parsed, canonicalized, and de-duped, over 80% can be matched. That is, canonicalization will be a key component of a name-based infrastructure.

Canonicalization may result in access to additional information; the canonical match of **Brucella abortus** to **Brucella abortus (Schmidt 1901) Meyer and Shaw 1920 (Approved Lists 1980)** provides access to authority information. Despite the improvement in matching, some caution is required. Canonicalization overcomes problems of variation in authority information but may lead to loss of taxonomic accuracy in matches (**Rattus rattus complex lineage III** matched **Rattus rattus (Linnaeus, 1758)**. Accuracy may be lost through elimination of some name elements (**Paludibacter propionicigenes CCUG 53888** being treated as the same as **Paludibacter propionicigenes**, and **Cucumis melo subsp. melo var. conomon** matching to **Cucumis melo**, and 40,000 or so name-strings that start with **Lepidoptera sp. BOLD** match to **Lepidoptera** in Catalogue of Life). Canonicalization may cause errors with symbionts: (**Melanocetus johnsoni symbiont** matches to **Melanocetus johnsoni (non Günther, 1864)**) and common names (**Cyprus processionary caterpillar** matched to **Cyprus)**. Canonicalization may remove some terms that either negate or clarify the name-string: **Acacia catechu auct. non Willd.**, matching to **Acacia catechu Willd**. The business rules of the underlying parser need to be editable so that solutions to unanticipated problems can be eliminated. The limitations may also be addressed through the match-scoring system, which can take into account differences in authorship, ranking, concept annotations, etc.

**Fuzzy matching.** Fuzzy matching tools were introduced to address variant spellings, mis-spellings or OCR errors (Rees 2014). Performance is about 80% satisfactory with an edit distance of ‘1’ - meaning that one in five matches is incorrect - **Calonectria microconidialis** matched to **Calonectria macroconidialis (Crous, M.J. Wingf. & Alfenas) Crous 1999**. Performance drops to an unacceptable 50% at greater edit distances (Fig. 5). A parsing step that assumes scientific names begin with a capital letter were the cause of failures to fuzzily match names from which the first letter missing (**olanum sendtnerianum Van Heurck & Muell.Arg.**). 

If fuzzy matching is to remain part of the tool kit (arguably it is valuable to help manage OCR errors) then improvements are needed. Firstly, the approach should be limited to canonical elements to eliminate the consequences of noise in author and date information. If edit distances greater than 1 are used, we should associate the more exacting result (**Chiatherina sp. ZSM 34143** matches **Iriatherina** at a distance of 2, and **Chilatherina** at a distance of 1) with the confidence score when fuzzy matching hits more than one target. Under those circumstances, additional semantic elements (perhaps species and subspecific names or authority information) may be called on to evaluate the competing matches. Knowledge of Latin and Greek grammar - such as gender compliance - may be used to discriminate among results: **Aphis citricida** matched to **Aphis citricidus**, **Aphis citricola Del Guercio, 1917**, and **Aphis citricola van der Goot, 1912.** Knowledge that -us and -a are likely to be interchanged as new combinations are formed would help to eliminate uncertainty. 

**Cross Mapping.** This is a useful tool that can have far-reaching benefits, especially in resolution services. The level of match between name-strings varies. At one end of the spectrum are instances in which all characters in a source name-string referring to a terminal taxon match a string in a target. Such matches may be the best, but could also be misleading. The increasing redistribution of digital names lists without any critical oversight may lead to matches among sources that have not verified the validity of the names. As an example, some recognize that the Global Names Index is not a source for taxonomically endorsed names, but others (e.g. http://marine.lifewatch.eu/belgian-lifewatch-e-lab) do treat it as a taxonomic database. Perfect matches may then be formed with other instances of the same mis-spelled name-strings or with chresonyms. Cross-checks against multiple taxonomically endorsed data sources or annotation (see below) are desirable to eliminate such matches. The next level of performance is a perfect match of the canonical versions of the terminal taxa. There is a small level of risk of homonyms (such as the examples of **Asterina gibbosa** and **Baileya australis** given above). Most homonyms have been listed in the IRMNG compilation and so can be converted into a reference vocabulary that the cross mapper can call upon to alert users to the possibility of a homonym. Less precise matching, from rank of genus and above is not useful if the agenda is to use names to interconnect data. Given the numbers of homonyms (McNeill 1997) canonical matches of generic names are likely to encounter homonym problems.

The errors that we detected with cross-mapping suggest that some improvement in business rules is required. Not only do we need devices to manage homonyms and chresonyms, but also to address name-strings that contain more than one taxon name. Such instances include hybrids, parasites, inquilines (**Nepenthes narrow-mouthed frog**) and other symbionts. Other problem areas include mapping common names to scientific names (**Crosse's shrew** matched to **Crossea**), and names with negating elements such as cf. and other negatives such as 'non', like, aff, nr. cf or sensu auctt. Following the current exercise, a new version (0.1.8) of the cross-mapping tool has been released with additional functionality to address issues with synonyms.

**A need for filters (vocabularies).** Expert sources are an essential source of information that can improve names services. The Global Names Architecture is seen as a system to draw on such sources to provide valuable services to the users of names. Such data can be used to disambiguate ambiguous results, filter or corroborate insights. Useful expert data that would be valuable include:

- Multiple taxonomic sources to capture a diversity of taxonomic views;
- Integration of nomenclatural registries because their focus on code-compliance will identify those names that are eligible as scientific names; and will help to eliminate chresonyms;
- Lists of scientific names that also occur in plain language dictionaries - such as *Bison*, *Cafeteria*, or *Torpedo* - so that names are not excluded simply because they occur within a plain language dictionaries;
- Lists of legal, medical, and other terms (such as Anorexia nervosa or Etcetera etcetera) that can be confused with scientific names;
- Common language dictionaries from the romance languages (French, Italian, etc.) that have many words that overlap with Latin terms;
- Compilations of common names that could be used to enhance reconciliation groups;
- Lists of homonymic taxa: these are already available from Interim Register of Marine and Nonmarine Genera (IRMNG), and can be used to annotate species or generic names that lack author information while being processed by GN tools to alert users of the need to be careful; species binomials that include a homonymic genus name that are not in authoritative lists should also be marked;
- Lists of names that are commonly misinterpreted by fuzzy matching - such as ‘hybrid’ being transformed into ‘hybrida’;
- Terms that negate the name (such as 'non', like, aff, nr. cf or sensu auctt.);
- Known parasites and symbionts to avoid **Phaseoulus vulgaris phytoplasma** being reported as a record of **Phaseoulus vulgaris**, rather than the **phytoplasm**; or terms that indicate an symbiotic association to better manage name-strings that relate to association such as **Vesicomya gigas endosymbiont**. This list would include terms such as ‘symbiont’, host’, ‘parasite’, or similar term; inclusion of ‘of’, ‘ex’ (also used with other meanings in name-strings) or ‘from’, or the use of inverted commas.
- Improved recognition of hybrids - inclusive of the mis-placement of the X sign to appear like an extension of a name.

### Open-ness, rights, and credit

Some expert sources of name-related information do not make their content openly and freely available, often using an argument based on copyright. Algaebase epitomizes the view that taxonomic content can be subject to intellectual property rights. At the time of writing, its website states:

“The images, information and data on this site are not in the public domain and are the property of the copyright owners. The data may not be downloaded or replicated by any means, manually or mechanically, including copying and pasting into theses, papers and other publications, and extraction by any means, manually or electronically. Any copying of the data or images, be it commercial or non-commercial (including non-profit), educational or non-educational, without the written permission of the copyright owner (generally AlgaeBase) and payment, if requested, may result in legal action, including legal action involving the service provider or publisher. See this site regarding copyright owner's rights. Fair usage of data in scientific publications is permitted, but not of images. ...All use, including all commercial or educational use and all use in web sites, whether public or private, is subject to copyright law worldwide. “

The site then provides a link to the US copyright law page.

The application of copyright law is not the same in different countries (Egloff et al. 2014). None the less, the US site states clearly that copyright applies to:

“(a) Copyright protection subsists, in accordance with this title, in original works of authorship fixed in any tangible medium of expression, now known or later developed, from which they can be perceived, reproduced, or otherwise communicated, either directly or with the aid of a machine or device. Works of authorship include the following categories:

(1) literary works;

(2) musical works, including any accompanying words;

(3) dramatic works, including any accompanying music;

(4) pantomimes and choreographic works;

(5) pictorial, graphic, and sculptural works;

(6) motion pictures and other audiovisual works;

(7) sound recordings; and

(8) architectural works.”

Copyright refers only to original works of creative art, not to data - such as the names and authors of taxa. That is, the data claimed to be under copyright by Algaebase is not so covered (Patterson et al. 2014). Rather, compilers of data who wish to restrict use of their content, can do so by applying ‘Data Use Agreements’. These can be used to impose limits and conditions upon data re-use.

Our belief is that the motivations behind this misleading copyright statement is a desire for credit and recognition for effort invested, to enable continued sponsorship. Our recommendation (Patterson et al. 2014) is an annotation system to ensure that re-use can be monitored, and the usage metrics be provided to sources and managers of names.

### Annotation

Annotation systems allow comments to be added to digital data objects. A generic system is hypothes.is. Two systems are being applied to Biology, Filtered Push and AnnoSys (Morris et al. 2013, Tschöpe et al. 2013). We see the ideal system as one in which each annotatable object is assigned a Universally Unique Identifier, and annotation tools in the form of plug-ins for browsers (see NameSpotter discussion below) allow comments to be added to the identifiers. Reconciliation is likely to be required for items that have more than oner UUID. The annotation tools might be activated by mouse-overs or embedded in specialist editing environments. Annotations, once made, will be then compiled centrally, can accompany the data object, be made visible to the data source or supplier of the digital object, and may or may not automatically update the digital object. Annotation can be used to correct errors or add additional information. It provides a mechanism for quality control. It is an appealing solution because quality control based on users will target content in use. In the case of a names-based infrastructure, annotation could be used to confirm or reject the results of fuzzy and canonical matching, address matters relating to homonyms, correctly identify synonyms and distinguish them from chresonyms, link or remove names in reconciliation groups, better manage common names, and so on. Given the inherently ‘dirty’ nature of biological data, we, like BiOnym (vanden Berghe et al. 2015​), feel that future workflow needs a combination of algorithmic approaches and expert human intervention.

### Future proofing the usefulness of names as metadata

A number of developments would improve the usefulness of names in publications or other electronic sources so they are better fitted to a role in indexing and managing distributed data.

The first element would be an open and highly visible tool based on the Global Names Recognition and Discovery algorithmcapable of identifying names in many formats such as text documents, pdf files, spreadsheets, lists, and images. Its role would be to recognize familiar name strings, their variant forms, or discover unfamiliar name-strings. It would then report if the name is known to preferred taxonomic authorities, if it is a senior synonym, if it is spelled correctly, if it has the right authority information, or if it needs to be updated. The NameSpotter extension of GNRD demonstrates that anchors can be added to name-strings in sources, and outbound links added to make the names in documents actionable. Such actions may access data from other sources, confirm if the spelling is correct, that the authority information is correct, or if the name is the senior synonym according to a preferred taxonomic source.

The second component is to add UUIDs to name-strings and/or to register identifiers if they already exist. UUIDs are globally unique, can be dereferenced in perpetuity to access the data that the identifier refers to, and are standardized for the discipline (Guralnick et al. 2015). UUIDs allow differences not immediately evident to become clear. *Homo sapiens* (UUID 16f235a0-e4a3-529c-9b83-bd15fe722110) and *Homo sаpiens* (UUID093dc7f7-5915-56a5-87de-033e20310b14) have different UUIDs because one example uses a Cyrillic ‘а’ character that looks the same as a latin ‘a’ character. UUIDs that are derived algorithmically from the string reveal the difference. 

A URI (a pointer to a location accessible through the Internet coupled with a UUID, such as urn:lsid:zoobank.org:act:EF59CD8D-2E6A-4B23-B9FB-DA6B3AC0A7F9) is seen as a good though not flawless solution (https://plus.google.com/+GregorHagedorn/posts/Q3vhs6pZCa). The use of a shared algorithm to generate the same UUID for a name-string enables data providers to locally mint the same identifiers for identical strings and avoid dependence on services (https://github.com/GlobalNamesArchitecture/GlobalNamesArchitecture.github.io/blob/master/_posts/2015-05-31-gn-uuid-0-5-0.md). However, our preference is for all appropriate GN services to use UUIDs or attach UUIDS to name-strings bereft of them, and be able to report the original names with the correctly spelled senior synonyms, and include a URI link to the name and through it to further information at other expert sites.

The third element is to embed the UUIDs in reconciliation groups. With this in place, name-strings in static documents can be updated at any time in the future to correct for future discovery of errors such as spelling errors or authority information; or if the scientific name is rendered into synonymy. Plugins can replace obsolete names with current ones, and names in documents could be made actionable through links to remote information.

A fourth component is an annotation system that enables users to comment on all name-strings in use, correcting any errors, adding information if absent, and otherwise improve the quality of the names environment.

The use of UUIDs and annotation has the benefit that appropriately designed plugins can be used to track and report the movements of name-strings from sources to users, and their subsequent re-use. This will create usage metrics, and these can be reported to provide credit to the creators and curators of names, indeed anyone who plays a key role in making names available and ensuring the quality of on-line services.

As for future practices, users should adopt scientific names where possible for terminal taxa. Authors should limit themselves to canonical versions of names, given that the data on authors and dates are not reliable. Exceptions may be needed with homonyms. Common names and taxonomically imprecise names should not be used. If no name is available, the authors should obtain voucher material and use a name-string that is linkable to other sources of information so that, in the future, they can call on new information about the taxon. Authors should have access to validation tools that confirm spelling, that the name is endorsed by a taxonomic authority (and if it is not, report the senior synonym if known), and finally to alert the user if the name is a homonym. The validation tools should add URIs to the names.

## Figures and Tables

**Table 1.** Revised profile of composition of name-strings in GenBank and DRYAD (DRYAD name-strings have been pre-processed), values are percentages of unique name-strings.

**Class**	**GenBank**	**DRYAD**
Species	40.1 %	82.3 %
Infraspecies	2.2 %	0.9 %
Genera	28.7 %	10.1%
Higher taxa	13.7 %	5.9 %
Common	2 %	0.8 %
Not useful	5 %	0 %
Hybrids	0.1 %	0 %
Symbionts	0.3 %	0 %
Viruses	7.9 %	0 %

**Table 2.** Impact of pre-processing (right) on the composition of the body of name-strings from DRYAD as percentages of unique name-strings. Name-strings were assigned to classes algorithmically and then reviewed and corrected manually.

**Class**	**Verbatim**	**Pre-processed**
Species	71.8 %	82.3 %
Infraspecies	2.2 %	0.9 %
Other scientific names	15.9 %	16 %
Common	1.4 %	0.8 %
Acronyms	4.4 %	0 %
Other names	4.3 %	0 %

**Table 3.** Extent of identifiable overlap among data sources shown as a percentage of all unique canonical terminal taxa in the first named source.

	**species**	**infraspecies**	**unmatched terminal** **taxa**
**GenBank vs Catalogue of Life**	75.4 %	6.7 %	17.9 %
**DRYAD verbatim vs Catalogue of Life**	31.3 %	2.8 %	66 %
**DRYAD pre-processed vs Catalogue of Life**	92.1 %	2.1 %	5.8 %
**DRYAD verbatim vs GenBank**	25 %	1.4 %	73.5 %
**DRYAD pre-processed vs GenBank**	97.8 %	2.2 %	0 %
**DRYAD pre-processed vs GenBank vs Catalogue of Life**	91.9 %	8.1 %	0 %
